# Demic and cultural diffusion propagated the Neolithic transition across different regions of Europe

**DOI:** 10.1098/rsif.2015.0166

**Published:** 2015-05-06

**Authors:** Joaquim Fort

**Affiliations:** ICREA/Complex Systems Laboratory and Physics Department, E.P.S., University of Girona, 17071 Girona, Catalonia, Spain

**Keywords:** Neolithic transition, fronts, cultural transmission

## Abstract

The Neolithic transition is the shift from hunting–gathering into farming. About 9000 years ago, the Neolithic transition began to spread from the Near East into Europe, until it reached Northern Europe about 5500 years ago. There are two main models of this spread. The demic model assumes that it was mainly due to the reproduction and dispersal of farmers. The cultural model assumes that European hunter–gatherers become farmers by acquiring domestic plants and animals, as well as knowledge, from neighbouring farmers. Here we use the dates of about 900 archaeological sites to compute a speed map of the spread of the Neolithic transition in Europe. We compare the speed map to the speed ranges predicted by purely demic, demic–cultural and purely cultural models. The comparison indicates that the transition was cultural in Northern Europe, the Alpine region and west of the Black Sea. But demic diffusion was at work in other regions such as the Balkans and Central Europe. Our models can be applied to many other cultural traits. We also propose that genetic data could be gathered and used to measure the demic kernels of Early Neolithic populations. This would lead to an enormous advance in Neolithic spread modelling.

## 1. Introduction

The Neolithic transition is the shift from hunting–gathering into farming and stockbreeding. The dynamics of this major transition in human prehistory is very well known in Europe and the Near East, because in this area hundreds of Early Neolithic sites have been dated. The oldest Neolithic sites are located in the Near East. From there, the Neolithic spread westwards and northwards across Europe. There are two main models of the Neolithic transition in Europe. The demic diffusion model assumes that farming spread due to the migration of farmers into new regions [1], whereas the cultural model assumes that hunter–gatherers (HGs) learnt agriculture from neighbouring farming populations [2]. Some authors have argued for the importance of both demic and cultural diffusion, and pointed out that they might have dominated the process in different regions [3]. However, although demic mathematical models have been compared to archaeological data since long ago [4], no mathematical models have been applied to disentangle the importance of demic and cultural diffusion in different regions of Europe. This is the aim of this paper.

Recently, cultural transmission (i.e. HGs learning agriculture from farmers) has been combined with demic diffusion (i.e. farmers moving to new locations) [5]. The new demic–cultural model was compared to the observed average speed of the Neolithic front across Europe [6], and the percentages of demic and cultural diffusion at the continental scale were estimated [5]. However, rather than understanding just a continental average, the really interesting question is to analyse differences in speed in different regions in Europe, and use such differences to determine whether demic or cultural dispersal was responsible for the spread of the Neolithic in each region. Here we tackle both problems.

## 2. Results

**Figure 1.**
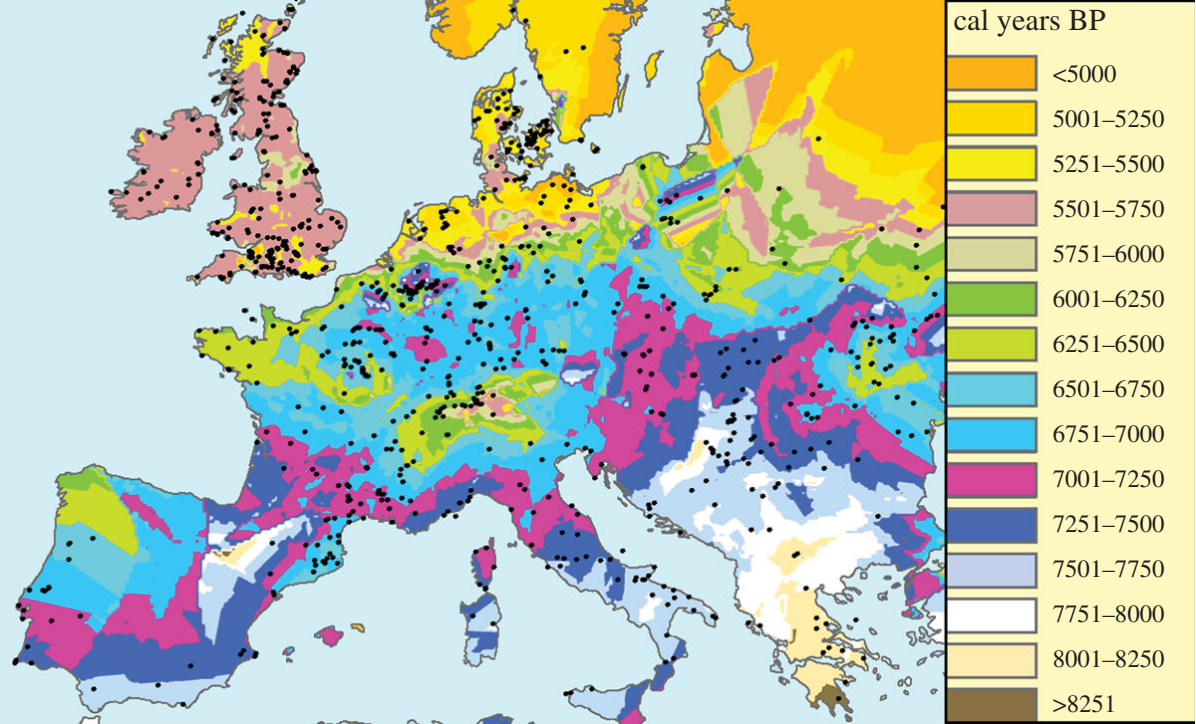
Isochrone map. The spread of the Neolithic transition, obtained by interpolating the dates in calibrated years before present (BP) of 918 Early Neolithic sites (circles) in Europe and the Near East (see the electronic supplementary material for details on the dataset and interpolation)*.* Map created with ArcGIS 10.

**Figure 2.**
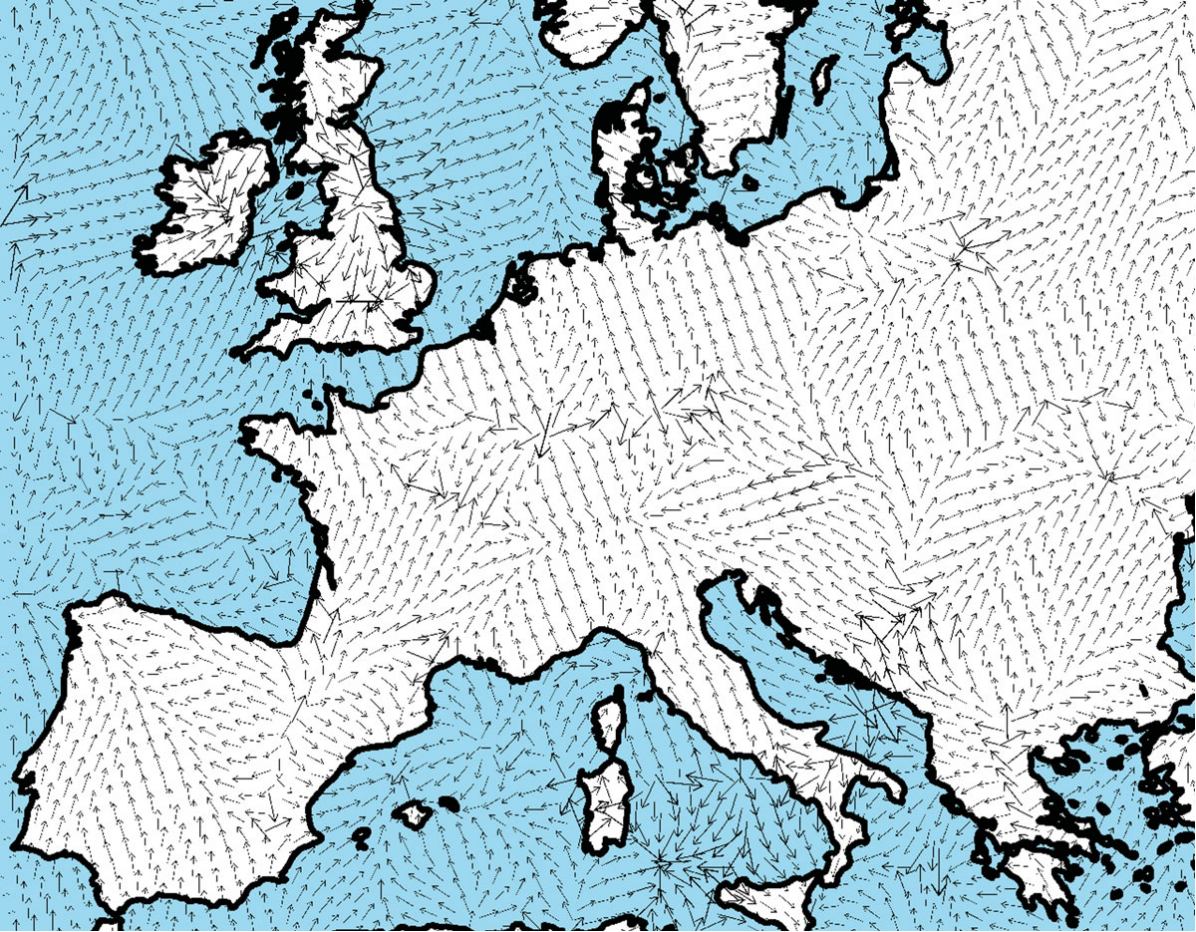
Directional map of the Neolithic wave of advance. Local speed vectors are shown. The corresponding local speed magnitudes, computed along the front propagation direction, are shown in figure 3. Map created with ArcGIS 10 and the Spatial Analyst extension.

**Figure 3.**
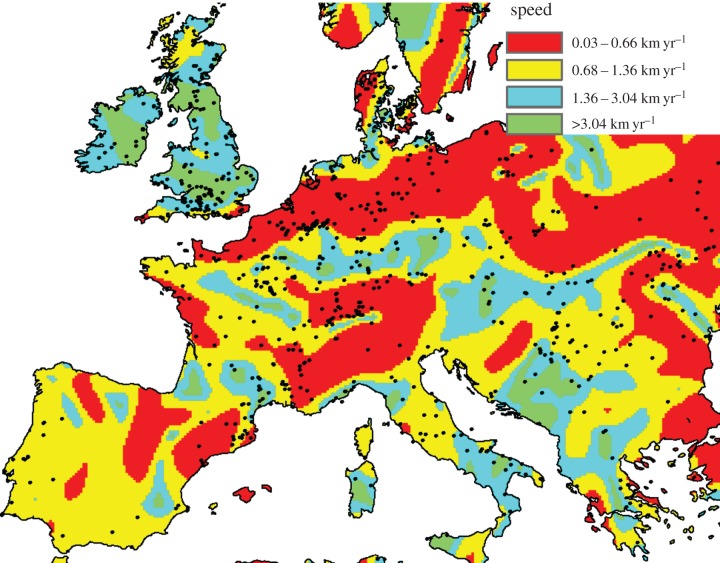
Demic versus cultural diffusion. In the red regions, the Neolithic spread was slow and due to cultural diffusion. In the yellow regions, the spread was faster and dominated by demic diffusion. In the blue regions, the speed was still faster and either demic or cultural diffusion could have dominated (this conclusion is due to parameter uncertainty). Note that some regions contain none or just a few sites and are thus highly uncertain, e.g. the yellow/blue/green area in Belorussia (upper right) and some scattered continental areas with very fast speeds (green). Map created with ArcGIS 10 and the Spatial Analyst extension.

We interpolated the calibrated dates [7] of 918 Early Neolithic sites in Europe and the Near East (Methods). In this way, we obtained the isochrone map in figure 1. Each colour corresponds to a 250-year time interval for the arrival time of the Neolithic. In figure 1, we can see the slowdowns in the Alps and Northern Europe (where successive isochrones are closer). In contrast to isochrone maps (such as figure 1), maps of the Neolithic speed magnitude have not been previously produced. Our first aim is precisely to compute such a speed map, from the isochrone map in figure 1. This is not straightforward because figure 1 has many small, closed areas of a colour different from that of their surroundings. This is due to the presence of sites that are substantially older or younger than nearby ones, and leads to abrupt changes in speed magnitude and direction (electronic supplementary material, figure S1*b*,*c*). For this reason, in order to estimate local front speeds, it was necessary to apply a smoothing procedure to figure 1. It simply averages the dates over nearby values. Without neglecting any dated site, this smoothing procedure removes artificial variations due to dating errors, calibration errors, etc., and produces realistic speeds in spite of the fact that not all sites have been discovered and dated, possible secondary diffusion effects, etc. (Methods and electronic supplementary material, text and figures S1–S4). In this way, we obtained the speed direction map in figure 2 and the speed magnitude map in figure 3. According to figure 3, the Neolithic spread was clearly slower in Northern Europe and the Alps than in the rest of Europe. We also note that the results in some regions are probably not reliable due to the lack of sites. For example, in Iberia (just below the central Pyrenees) there is an anomalously old region according to figure 1 (see also electronic supplementary material, figure S3). This region leads to an apparent Neolithic source in the arrow map in figure 2, surrounded by two apparently slow (red) regions in figure 3. Similarly, there is an anomalously old (white) region in Croatia (figure 1). Such results are very likely to be artefacts, because unfortunately there are almost no dated sites in both anomalously old regions (figure 1). More complete datasets may in the future solve such local problems. However, in most regions of Europe, there are many sites per unit area, so additional sites will presumably lead to small changes in most areas. Most importantly for the purposes of this paper, the slowdowns in Northern Europe and the Alps are reliable features, because there are many sites in these regions (figure 3) and these slowdowns are also apparent from the isochrone map (figure 1). The dating uncertainties of the archaeological sites do not affect this conclusion (electronic supplementary material). We next combine these empirical findings with mathematical models, in order to analyse the implications on the human behaviour at the onset of the Neolithic in different regions.

### 2.1. Purely demic model

Several purely demic models have been proposed. The simplest one is given by Fisher's equation [8]2.1
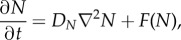
where *N* is the population density of the Neolithic population (number of farmers per unit area), *D_N_* is its diffusion coefficient, and *F*(*N*) = *a_N_N*(1 − *N*/*K_N_*) is the logistic function describing net reproduction (with *a_N_* the initial growth rate and *K_N_* the carrying capacity or maximum population density). *D_N_* is a measure of the mobility of the population during a generation time *T* (defined as the mean time interval between the migration of an individual and one of her/his children [9]). Logistic growth functions are well known to agree with many population data for humans (see citations in [10]).

In Fisher's model, changes in the local population density of famers (left-hand side in equation (2.1)) are due either to farmers moving around (first term on the right-hand side of equation (2.1)) or to their net reproduction (last term in equation (2.1)). There are no HGs becoming farmers in this model, so agriculture spreads due only to the diffusion of agricultural populations. It is thus a purely demic model. Front solutions to equation (2.1) propagate with Fisher's speed [8]2.2
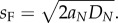


In recent years, it has been shown that the approach based on equations (2.1) and (2.1) is not accurate for human populations [11,12] and that a realistic, reaction-dispersal cohabitation model leads to the following speed rather than equation (2.1) (see electronic supplementary material for a derivation)2.3
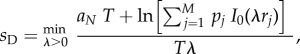
where *p_j_* is the probability that an individual disperses at distance *r_j_* (*j* = 1, 2, … ,*M*) (i.e. the demic kernel). 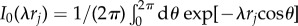 is the modified Bessel function of the first kind and order zero. The sub-index *D* in the left-hand side of equation (2.3) indicates that this is a purely demic model. We have used equation (2.3) with dispersal kernels obtained from ethnographic observations of five preindustrial farmer populations and realistic ranges for *a_N_* and *T.* This yields the range 0.68 < *s*_D_ < 1.48 km yr^−1^ for the speed according to the purely demic model (electronic supplementary material). We stress that we have applied equation (2.3) rather than Fisher's equation (2.2) because the latter is based on ordinary diffusion, an approximation that leads to very large errors (electronic supplementary material, Ordinary diffusion section), especially if cultural transmission is included (next paragraphs).

### 2.2. Demic–cultural model

We extend the model above by including cultural transmission, i.e. by taking into account that farmers may teach agriculture to HGs living at some distance. In this new model (electronic supplementary material), the speed of the Neolithic front is2.4
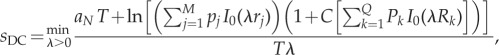
which generalizes equation (2.3) by means of the parameter *C* (the intensity of cultural transmission) and the probabilities *P_k_* that a HG learns agriculture from a farmer living at distance *R_k_* (*k* = 1, 2, … ,*Q*) (electronic supplementary material). Note that this cultural kernel {*P_k_*, *R_k_*} is different from the demic kernel {*p_j_*, *r_j_*} in equation (2.3). The sub-index *DC* in the left-hand side of equation (2.4) indicates that this is demic–cultural model. Without cultural transmission (*C* = 0), equation (2.4) reduces to equation (2.3), as it should.

For given parameter values, the demic–cultural model (equation (2.4)) will always lead to a faster Neolithic front than the purely demic model (equation (2.3)). The intuitive reason is that additional farmers result from the acculturation of the HG population, and this will propagate agriculture faster. Obviously, the minimum speed of this demic–cultural model (equation (2.4)) is the same as for the purely demic model (equation (2.3)), namely 0.68 km yr^−1^ (*C* = 0). Thus, the relevant result from the demic–cultural model is its maximum possible speed. This turns out to be *s*_DC max_ = 3.04 km yr^−1^ (electronic supplementary material) according to equation (2.4) with the observed range of the cultural transmission intensity *C* and the observed cultural kernels for five populations of HGs who learn agriculture from farmers (as well as the parameters already used in the purely demic model above).

From figure 3, we note that in some regions (Northern Europe, the Alps and west of the Black Sea), the observed speed is inconsistent with both the demic model (equation (2.3)) and the demic–cultural model (equation (2.4)), because both of them predict very fast speeds (greater than 0.68 km yr^−1^) when compared with those implied by the archaeological data in those regions (red colour in figure 3). Clearly, a different model is necessary to describe the spread of the Neolithic in those areas. Therefore, we next propose a purely cultural model.

### 2.3. Purely cultural model

The observed slow speeds in, e.g., Northern Europe and the Alps (red areas in figure 3) were not detected in [5–7], because those works computed only an average, global speed over all of Europe (not a map of the speed as a function of position, as in figure 3). As explained in the previous paragraph, in order to explain speeds below 0.68 km yr^−1^ we clearly need a purely cultural model, which has not been considered previously. Obviously, a purely cultural model means no demic diffusion. In this model (see the electronic supplementary material for details), the front speed can be obtained from equation (2.4) without demic diffusion (*r*_1_ = 0 and *p*_1_ = 1), namely2.5
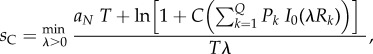
where the sub-index *C* in the left-hand side indicates that this is a purely cultural model (no demic diffusion). In this model, there is no occupation of new areas by farmers neither converted HGs, but only conversion of HGs into farmers (cultural diffusion) at distances *R_k_* with probabilities *P_k_*. It is reasonable to argue that real models will never be purely demic or purely cultural. Certainly, both extreme cases should be considered only as approximations. But they may provide reasonable descriptions of the propagation of the Neolithic wave of advance in some regions (and are simpler to apply that more detailed models). For example, the assumption behind equation (2.5) is that dispersal of children away from their parents has a negligible effect on the front speed. However, adding population dispersal explicitly to equation (2.5) is possible by means of a short-distance dispersal kernel for HGs converted to agriculture and their descendants (in areas without incoming farmers). This complicates the estimation of the predicted speeds (electronic supplementary material) but does not change the conclusion of this paper (see the results below).

Using equation (2.5) with the same observed cultural kernels and parameter values as above, the purely cultural model yields the speed range 0.03 < *s*_C_ < 0.66 km yr^−1^ (electronic supplementary material).

### 2.4. Demic versus cultural diffusion on a map of Europe

In figure 3, the colour scale has been chosen so that the red colour corresponds to the regions of purely cultural diffusion (0.03–0.66 km yr^−1^, from the purely cultural model above). The demic and demic–cultural models predict speeds above 0.68 km yr^−1^ and are thus too fast to be consistent with the archaeological data in the red regions in figure 3. Thus, we conclude that cultural diffusion explains the Neolithic transition in Northern Europe, as well as in the Alps and west of the Black Sea.

The analysis of the areas where demic diffusion played a role is less straightforward, because any speed in the range 0.68–1.48 km yr^−1^ is consistent with purely *demic* diffusion (see above), i.e. *C* = 0 with appropriate parameter values, but it will also be consistent with the demic–cultural model with, e.g., a lower value of *a_N_* and a value of *C* ≠ 0 (because decreasing *a_N_* decreases the front speed, whereas increasing *C* increases it). Thus, due to parameter uncertainty, any given speed in the range 0.68–1.48 km yr^−1^ is consistent with many different realistic sets of values for *a_N_*, *C*, *T*, {*p_j_*, *r_j_*} and {*P_k_*, *R_k_*}, and therefore with many different possible percentages of the cultural effect. But if we consider, e.g., a speed of 0.70 km yr^−1^, the cultural effect will be surely very small (because demic diffusion is responsible for at least 0.68 km yr^−1^). Reasoning in this way, it is possible to determine the regions where the speed was mainly demic, i.e. where the cultural effect was less than 50% (Methods), and they correspond to the yellow regions in figure 3. The regions where either demic or cultural diffusion could have dominated are the blue regions in figure 3 (Methods). Finally, in the green regions in figure 3 the speed is too fast to agree with any of the three models in this paper, but in continental Europe those regions contain very few sites, so they are probably statistical artefacts (they will likely disappear when more sites are discovered and dated).

As explained above, for mathematical simplicity the purely cultural model (equation (2.5)) neglects the dispersal of HGs converted into farmers, but it can be included by means of a short-range dispersal kernel (electronic supplementary material). The assumption that the dispersal kernel for converted HGs and their descendants has a short range seems very reasonable because both ethnographic [13–15] and genetic [16,17] data indicate that partially converted HGs have substantially lower average dispersal distances. Including a dispersal kernel of converted HGs and their descendants still leads to a purely cultural model (not a demic–cultural one) because the genes (and possibly the language) of converted HGs will be those of HGs, not those of farmers (a more detailed discussion on this point is included in the electronic supplementary material). Obviously, adding the effect of a dispersal kernel to that of a cultural one will lead to a faster front. For this reason, the speed ranges from the purely cultural and the demic–cultural models may overlap and this may make the dominant model of spread uncertain in some regions (compare the blue regions in figure 3 (model without dispersal of converted HGs) and electronic supplementary material, figure S5 (model with short-range dispersal of converted HGs)). Therefore, depending on the dispersal kernel of converted HGs, inference on the dominant mode of dispersal might not be possible in some areas. However, the regions where we can safely conclude that cultural diffusion dominated (red) are the same in both models (figures 3 and electronic supplementary material, figure S5). Thus, inclusion of a short-range kernel for converted HGs does not change our main conclusion (namely, that cultural diffusion explains the Neolithic transition in Northern Europe, as well as in the Alps and west of the Black Sea).

In the British Islands, the speeds are very fast (figure 3), as previously noted by Bocquet-Appel *et al.* [18] by a different approach (namely, using the average of the two earliest dates in each square of 35 × 35 km [19] rather than smoothing over all sites as in this paper). In figure 2, we can also see a dual entrance into England, from the south and from the north, which has been previously observed in a set of maps of the spatial density of calibrated dates separated 100 years [20]. Henderson *et al.* [21] also followed a different approach by fitting a uniform background speed (a fixed value for all of Europe) plus additional speeds due to several rivers/coasts (a fixed speed for each river/sea). They, as well as previous authors [4], noted the fastness of the spread between the slow (red) regions in Northern Europe and the Alps in our figure 3, and this feature will be interpreted in the next section (in connection with the Linearbandkermic (LBK) culture). For the red region in Northern Europe in our figure 3, we cannot compare to Bocquet-Appel *et al.* [18], because they reported the speed over a wider region to the north and east (which are substantially faster, see the isochrones in their fig. 1). For this reason, the slow speeds in Northern Europe, the Alps and the Black Sea (red regions in figure 3) cannot be compared to the estimations by Bocquet-Appel *et al.* [18], due to the fact that they did not report speeds locally but over wide regions (the eight regions in their fig. 2). However, the slowness in the three red regions in our figure 3 is seen qualitatively in their isochrone map (fig. 1 in [18]). Thus, their results are qualitatively consistent with ours, in spite of using different methods (see above). The main differences those previous papers [18,19,21] and the present one are: (i) we have produced a map of local speeds (figure 3), not estimations over wide areas, and (ii) we have interpreted the local speeds in terms of demic and cultural diffusion using mathematical models.

## 3. Discussion

Mathematical models make it possible to obtain maps of demic versus cultural diffusion (figure 3) solely from the dates of archaeological sites (we have not used genetic neither any other kind of data). Thus, the methodology introduced in this paper makes it possible to identify regions where demic or cultural diffusion dominated, using only archaeological data.

The Neolithic transition in Europe was a complex, highly non-homogeneous process, and took place under the following scenario according to the methodology in this paper. The Neolithic arrived in many regions of Europe mainly via demic diffusion (yellow in figure 3), or possibly mainly either demic or cultural diffusion (blue in figure 3). This process was fast (speeds above 0.68 km yr^–1^) and includes the Greece, Italy, the Balkans, Hungary, Slovakia, Czechia and central Germany. We note that this includes a substantial part of the LBK culture in Central Europe (fig. 12.7 in [22]). This agrees with the fact that the LBK is widely regarded as demic by archaeologists, because of the observed discontinuities between HGs and farmers (in house forms, settlement patterns and stone tool types), the geographic cultural uniformity of farmers (in pottery, house forms and settlement location) over huge areas [23], and the rapid rises in population numbers [24]. Some archeologists have also argued for the importance of demic diffusion in the Neolithic spread from the Aegean northwards and across the Balkans [22] and this is also in agreement with our results (which imply that demic diffusion played a role in the yellow and blue regions in figure 3).

Our models also suggest that farming populations did not spread effectively into regions such as Northern Europe, the Alps and west of the Black Sea (red colour in figure 3). There the transition was slow (speeds below 0.66 km yr^−1^) and not driven by demic or demic–cultural diffusion (according to the models analysed). Based on observations of continuity in culture and settlement location, some archaeologists have previously suggested that cultural diffusion had a strong role in the spread of the Neolithic in Northern Europe [22,25,26], the Alps [25,27] and west of the Black sea [28]. Interestingly, these are precisely the mainly cultural diffusion regions according to our mathematical models and estimated front speeds (red colour in figure 3). Some researchers have also proposed several possible explanations. For example, perhaps higher HG densities and/or more productive ecosystems for hunting and/or gathering acted as barriers to the demic diffusion of farmers [29]. Or maybe the time needed for some crops to gradually adapt to a different climate [30–31] allowed for more time for HGs to gradually familiarize with agriculture. Or perhaps a combination of these (and possibly other) factors may explain the differences between the importance of demic and cultural diffusion in different regions. The models and results in this paper do not assume (neither contradict) any of those possibilities. Our aim here is only to distinguish regions of mainly demic diffusion from regions of mainly cultural diffusion (figure 3). In order to do so, there is no need to assume any specific explanation for the existence of such regions. As seen above, it is sufficient to develop three different models (purely demic, demic–cultural and purely cultural) and compare their results to the observed speed map according to the archaeological data.

It could be argued that, alternatively, the slow speeds in the red regions in figure 3 might have been also due to a (mainly) *demic* spread (with a sufficiently slow reproduction rate or/and a sufficiently narrow demic kernel). Such a view would advocate for a mainly demic spread over all of Europe. By contrast, according to our model, in the red regions in figure 3 the spread was (mainly) *cultural* (i.e. demic diffusion was negligible there). An additional argument for our proposal (besides the archaeological observations above [22,25,26]) is that the frequencies of Neolithic genes are higher in the Mediterranean than in Northern Europe (see the two regression lines in [32], figure 2). This has been interpreted by geneticists as evidence that *cultural* diffusion was more important in *Northern* than in Southern Europe [32]. This agrees with our model (figure 3). By contrast, the alternative view that the spread was mainly demic *both* in Southern and in Northern Europe is unable to provide an explanation for the observed genetic differences between Southern and Northern Europe. In fact, evidence for a higher importance of cultural diffusion in Northern Europe has been found not only in DNA data from present-day humans [32] but also from ancient humans [33] and from ancient pigs [34].

Genetics could provide very useful, direct measurements of prehistoric demic kernels, {*p_j_*, *r_j_*}, by identifying parent–child pairs and measuring the distances between them. If demic kernels displayed shorter distances in the red regions in figure 3 (mainly cultural diffusion according to our model) than in the rest of Europe, this would provide support for our model. Apparently such kinds of genetic data have not been yet gathered (because no Early Neolithic parent–child pairs have been identified so far), but they would be extremely useful. Indeed, using the observed demic kernel (as implied by them) into the mathematical models in this paper could definitely establish which of the views in the previous paragraph is consistent with the observed speeds.

Parameter estimation is uncertain not only concerning the kernels. Neolithic growth rates could also depend on the dominant mode of Neolithic spread (demic or cultural). Indeed, it is reasonable to expect that probably the converted HGs are not the best farmers yet, and this might have an effect on reproduction and/or survival of these individuals and their children. For example, it is known that the birth spacing in HGs is longer (and the mean number of children therefore lower) than in farmers [2]. Thus, partially or recently converted HGs could have lower reproduction rates than farmers. This is therefore another possible mechanism (besides narrower demic kernels) that could lead to slower front speeds in mainly cultural regions.

The parameter uncertainties explained in the previous paragraphs imply that our models can only lead to preliminary conclusions until the kernels, growth rates, etc., are accurately measured.

To some extent, our proposal could be also tested by purely archaeological means. Strontium isotope data have been often used to distinguish non-local from local individuals in cemeteries [35,36]. If such data showed a substantially lower mobility of Early Neolithic individuals in regions where cultural diffusion was responsible for the spread of farming (red regions in figure 3), when compared with the rest of Europe, this would provide support for our model. Although the genetic approach explained in the previous paragraph should be able to determine intergenerational distances with more precision than strontium isotope analysis, because the latter cannot determine exact distances but only distinguish local from non-local individuals [36], both approaches could complement each other and strengthen the conclusions.

If sufficient archaeological sites are dated in the future for the Neolithic transition that spread from the Near East into Asia and Northern Africa, as well as for Neolithic transitions in other continents, our methodology could be applied to determine the importance of cultural and demic diffusion in different regions.

Finally, let us note that the mathematical models proposed do not assume that the cultural trait that spread was agriculture. For this reason, these models can be applied to the geographical spread of any cultural trait, such as other transitions in human history, technological innovations, languages, etc., making it possible to determine the importance of demic and cultural diffusion in different regions (the only condition is the existence of sufficient data to estimate local front speeds, as well as of independent observations to estimate the values of the demic and cultural kernels and other parameters in the models). Some specific examples to which our models could be applied are the spreads of horse domestication in Eurasia [37], dairying in Europe [38], maize in America [39] and military technologies in the Old World [40].

## 4. Methods

### 4.1. Isochrone and speed maps

After trying a variety of interpolation techniques, we applied universal linear kriging to the calibrated dates of 918 sites and obtained figure 1 (see the electronic supplementary material for details on the database and interpolation techniques). In fact, universal kriging is widely recognized as the best interpolation method when there is a spatial trend in the data [41,42] (in our case, the trend is due to the spread of farming from the southeast). A map of isochrones (figure 1) is equivalent to a curved surface, with vertical coordinate equal to the interpolated arrival date. This defines a surface of interpolated arrival dates of the Neolithic. The original interpolation surface in figure 1 has many local maxima and minima and leads to abrupt changes in speed magnitude and direction (electronic supplementary material, figure S1*b*,*c*). For this reason, it was necessary to apply a smoothing procedure to figure 1 that simply averages over nearby values (for details, see the electronic supplementary material, figures S1–S4 and text). This leads to the speed vector and magnitude maps in figures 2 and 3.

### 4.2. Demic versus cultural diffusion map

(1) Using the observed cultural kernels and ranges for *a_N_*, *T* and *C* into equation (2.5) (purely cultural model) yields 0.03–0.66 km yr^−1^ (electronic supplementary material). This range is not consistent with either the demic or the demic–cultural models (0.68–1.48 km yr^−1^ and 0.68–3.04 km yr^−1^, respectively, see Results). It thus corresponds to purely cultural diffusion regions (red colour in figure 3).
(2) The range 0.66 < *s*_obs_ < 0.68 km yr^−1^ is not consistent with any of our three models, but it is so narrow that it is not seen in figure 3. Thus we feel it unnecessary to analyse the parameter ranges in depth in order to solve this negligible discrepancy.
(3) The analysis of speeds *s*_obs_ ≥ 0.68 km yr^−1^ is not straightforward. Here the purely cultural model (equation (2.5)) is too slow (see above). Hence we have to apply the demic–cultural model (equation (2.4)). As in [5], the cultural effect *E* (in %) is defined as the contribution to the observed speed that is due to cultural transmission4.1
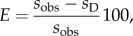
where *s*_obs_ is the observed speed at a given location in Europe (i.e. as estimated from the archaeological data) and *s*_D_ is the speed according to the purely demic model (for a detailed justification of equation (4.1), see electronic supplementary material). Within the purely demic model, the minimum speed is *s*_D_ = 0.68 km yr^−1^ (see above). Therefore, equation (4.1) implies that the maximum cultural effect *E* is attained for *s*_D_ = 0.68 km yr^−1^,

4.2
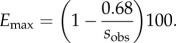

For speeds greater than or equal to 0.68 km yr^−1^, we consider the following cases.
  (3.1) If *s*_obs_ < 1.36 km yr^−1^, then *E*_max_ < 50%. These are thus mainly demic regions. They are shown as yellow in figure 3 (0.68–1.36 km yr^−1^). Note that, since 0.68 < s_D_ < 1.48 km yr^−1^ (see *Results*, purely demic model), in this case *E*_min_ = 0%.
  (3.2) If *s*_obs_ ≥ 1.36 km yr^−1^, then *E*_max_ > 50%. However, we cannot assure that these are mainly cultural regions unless we can also show that *E*_min_ > 50%. According to equation (4.1), *E*_min_ is attained for the maximum value of *s*_D_, namely *s*_D_ = 1.48 km yr^−1^ (see above), 4.3
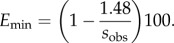

There are two important subcases, depending on whether *E*_min_ is less or more than 50%.
  (3.2.1) If *s*_obs_ < 2.96 km yr^−1^, then *E*_min_ < 50%. Thus either demic or cultural diffusion can dominate (blue regions in figure 3, 1.36–2.96 km yr^−1^).
  (3.2.2) If *s*_obs_ > 2.96 km yr^−1^, then *E*_min_ > 50%. Thus, cultural diffusion dominates also in these regions, i.e. they should also be red in figure 3. However, the maximum possible speed according to the demic–cultural model is 3.04 km yr^−1^ (electronic supplementary material), so this range (2.96–3.04 km yr^−1^) is in fact very narrow and, for this reason, it cannot be distinguished in figure 3.
    Note that the blue regions in figure 3 are ultimately due to the uncertainty in the parameter ranges. Indeed, if we had used a single value (not a range) for each of the parameters *a_N_* and *T*, and a single demic kernel, we would have obtained a single value for *s*_D_ (instead of the range 0.68–1.48 km yr^−1^). In such an instance, the calculation of mainly demic (*E* < 50%) and mainly cultural (*E* > 50%) regions would have been straightforward from equation (4.1), and no blue regions (where either demic or cultural diffusion can dominate) would have appeared in figure 3.
(4) Finally, if *s*_obs_ > 3.04 km yr^−1^ the observed speed is inconsistent with our models. However, there are only some small scattered areas with so fast speeds (green in figure 3) and are probably artefacts because they do not contain any or very few sites. The only exceptions are the British Islands, where the interpolation method is probably less reliable due to the lack of sites in the wide surrounding seas (similar to Denmark). Thus the analysis of such local features is deferred to future work.

## Supplementary Material

Supplementary Information Appendix

## Supplementary Material

Fig. S1

## Supplementary Material

Fig. S2

## Supplementary Material

Fig. S3

## Supplementary Material

Fig. S4

## Supplementary Material

Fig. S5

## Supplementary Material

Fig. S6
