# Supplementary material for: Demic and cultural diffusion propagated the Neolithic transition across different regions of Europe
Source: J R Soc Interface. 2015 May 6;12(106):20150166. doi: 10.1098/rsif.2015.0166 (PMC4424695; doi:10.1098/rsif.2015.0166)
Supplement: Supplementary Information Appendix [file rsif20150166supp1.pdf]

Supplementary Information Appendix

S1. Estimating the observed speed in different regions. ***Database.*** We used the dataset of 902 Early Neolithic sites in Europe from Ref. (1), where details on the data selection and calibration can be found. Because that dataset does not include Near Eastern sites, we complemented it with the 16 calibrated dates of PPNB/C sites in the Near East obtained from the CONTEXT database in Ref. (2). As explained in Ref. (2), we consider only PPNB/C sites in the Near East because it is from the PPNB/C cultures that a homogeneous set of farming practices spread into Europe (different Neolithic innovations appeared at different places and times in the Near East, and for this reason earlier Neolithic cultures were not part of the front that propagated into Europe), see specially Fig. 5 in (2) for a quantitative justification and further details.

Let us briefly explain why we have used all sites in the original databases (1,2), rather than keeping only the earliest regional sites. Obviously, keeping all sites in a region will lead to somehow more recent estimates for the arrival of farming than keeping only the oldest site(s) in each region. The existence of sites with different dates in a given region may be due to several reasons. For example, when the Neolithic wave of advance arrives into a region, farming will presumably begin in the most suitable areas for agriculture, and a secondary diffusion process towards less suitable areas (in several directions) may take place later on. However, in this paper we are interested in the front speed (not in the arrival time of farming) and, if all estimates for the arrival time are delayed by a similar number of years due to secondary diffusion, the inferred front speed will still be realistic (the same will happen if there is another homogenous, apparent delay due to the fact that not all sites have been discovered and dated). The existence of sites with different dates in a region may be also due to dating and calibration errors. However, this effect should gradually disappear by averaging over local sites, as done in this paper by means of a smoothing technique (details on that technique are given below). Thus in the present paper, we make use of all sites in the databases. Alternatively, it would be also reasonable to keep only the oldest Neolithic sites in each region and neglect the younger ones in it. However, such an approach has some problems. Firstly, it will tend to select sites for which dating errors have caused older dates. Secondly, if for example we divided Europe into squares of equal size and kept only one or two sites in each square (the oldest ones), different square sizes would in general lead to different results. Thirdly, different results would also follow depending on how many sites per square were kept. Fourthly, some squares would contain many sites and others would contain only one or a few, thus we would be neglecting many sites in some regions and (almost) none in other regions. In contrast, keeping all sites avoids dealing with those problems and making several assumptions (e.g., the shape, size and number of sites per region). Therefore, although it would be of interest to compare different approaches in future work, in this paper we have preferred not to neglect any site in the databases.

Interpolation isochrones and speed maps. Interpolation methods can be characterized as global or local (3,4). In global methods the result (interpolation) at each location is affected by all data, even those located very far away. In contrast, in local methods the interpolation at each location is computed using only nearby sites. We observed that global interpolation methods, e.g. polynomial surface interpolation (trend surfaces) (3,4) missed some important local features, such as the slowdowns in the Alps and near the Northern coasts of Europe. Thus we used a local method (universal linear kriging) to obtain the interpolation maps presented in the paper and this *SI Appendix*. Universal kriging is widely recognized as the best interpolation method when there is a spatial trend in the data (4,5) (in our case, the trend is due to the spread of farming from the Southeast). However, we noted that other *local* interpolation methods, e.g. ordinary circular kriging, natural neighbor, inverse distance weighting, etc. (3,4) yielded very similar maps to those presented.

Figure S1a is the result of interpolating our database (Fig. S1a here is the same as Fig. 1 in the main paper). Each color corresponds to a 250-y time interval for the arrival time of the Neolithic. In Fig. S1a we can see the slowdowns in the Alps and Northern Europe (because successive isochrones become closer). It will be useful to note that a map of isochrones (Fig. S1a) is equivalent to a curved surface, defined as follows. To each point on a horizontal map of Europe we assign a vertical coordinate (above the map) given by its interpolated arrival date. The date before present (BP) increases with decreasing vertical coordinate (i.e. older dates are nearer the horizontal map). This defines a curved surface of interpolated arrival dates of the Neolithic. The isochrones can then be viewed as the intersections of this surface with horizontal planes (i.e., planes of constant date BP). At each point of the surface, the gradient is a 2-dimensional vector with the following properties: (i) the gradient direction is that of maximum slope; (ii) the gradient magnitude is equal to the maximum slope; and (iii) the gradient is orthogonal to the corresponding isochrone (6). Obviously, the local front speed is also orthogonal to the isochrone. Thus the local speed vector is parallel to the gradient, and the front speed (measured in km/y) can be computed as the inverse of the gradient magnitude (measured in y/km). The geographical information software we have used finds out the direction and magnitude of the gradient (maximum slope) at each location of the map (7). By computing the inverse of the latter, we find the local front speed.

We first computed front speeds directly from the raw data. This yields the speed map in Fig. S1b. It poses the following problem. In previous works the average speed was estimated from linear regressions of distances from the Near East. That approach yielded an average speed (over all of Europe) of about 1 km/y (8,1,2). In contrast, the local speeds in Fig. S1b are much slower. The explanation is that the speeds in Fig. S1b are unrealistically slow, due to the fact that the interpolated surface (Fig. S1a) has many local maxima and minima, which also lead to unrealistically abrupt changes in speed orientation (Fig. S1c). We show both points in turn. Figure S2a is an enlarged region of the isochrone map (Fig. S1a). The ellipses indicate some examples of small regions with a color corresponding to a time interval older than that of the surrounding color. Obviously, if such regions were real features, agriculture would have

appeared in them by some non-local mechanism (i.e., from an older region not in contact with them, but located far away). This is one reason to suspect that such small regions are statistical artifacts. An additional argument is to note from Fig. S2a that these regions contain only one or very few sites (or even no sites at all). This shows that each such small region is due to the fact that a single site (or a few of them) is anomalously older than most of the surrounding sites. In contrast, when a large region contains many anomalously young/old dates (such as the Alps, i.e. the green and yellow areas in Fig. S2a), it is reasonable to assume that it corresponds to a real slowdown/acceleration of the Neolithic front. But small regions with almost no sites (ellipses in Fig. S2a) are most likely statistical artifacts (e.g., due to dating errors, calibration errors, etc.) or issues that can be disregarded for our purposes (e.g., secondary diffusion, the fact that not all of the sites have been discovered and dated, etc.), as discussed above.

We next justify that anomalously old/young regions will lead to abrupt changes in speed orientation and unrealistically slow speed magnitudes. For the sake of simplicity, let us consider an hypothetical example. Assume that in some region the 7,000-y BP and 6,000-y BP isochrones are separated about 1,000 km, so that in this hypothetical region the Neolithic front travelled at a speed of 1 km/y (Fig. S2b). Next assume that an anomalously early site were found between both isochrones, leading to the additional 8,000-y BP isochrone in Fig. S2c (i.e., to a small, anomalously old region similar to those inside ellipses in Fig. S2a). Then, the speed is much lower (0.2-0.4 km/y in Fig. S2c versus 1 km/y in Fig. S2b), and the speed vector changes abruptly its direction (compare again Fig. S2c to Fig. S2b). This clearly shows that anomalously old/young sites can seriously affect the computed front speed and its direction. Therefore, in order to take care of this effect, we need to smooth the small anomalously old/young regions (ellipses in Fig. S1a).

From a statistical perspective, a very reasonable way to smooth out anomalously old/young regions (which correspond to local minima and maxima on the interpolated surface, respectively) is to average each value with those at nearby locations. In fact, this approach is well-known in statistical spatial analysis. The computer program we used divides the geographical region (Europe and the Near East) into many small squares and replaces the value at each square by the average of this value and those at the 8 surrounding squares (7). Not surprisingly, performing this smoothing procedure only once results in an isochrone map (Fig. S3a) very similar to the original one (Fig. S1a). Thus, most anomalously old regions (e.g., those inside ellipses in Fig. S2a) still appear in Fig. S3a. However, by repeating this smoothing procedure several times, small regions shrink gradually (as we could expect intuitively). This is clearly seen by comparing the result of a single smoothing (Fig. S3a) with those after smoothing 10 times (Fig. S3b) and 20 times (Fig. S3c). Once most anomalously old/early regions have been smoothed out (see Fig. S4a, obtained after smoothing a total of 40 times), smoothing a similar number of times again (e.g., 20 more times, up to a total of 60 times) leads to no substantial changes in the speed magnitude (Fig. S4b) and orientation (Fig. S4c). Thus these are our final results for the observed isochrones and speeds, i.e. Figs. S4c and S4b are the same as Figs. 2 and 3 in the main paper, respectively. As expected from the hypothetical example in Figs. S2b-c, we see that the speeds

(Fig. S4b) are faster than before smoothing (Fig. S2b), and their directions (Fig. S4c) change much less abruptly than before smoothing (Fig. S1c).

S2. Models. ***Purely demic model.*** This model has been considered previously (9-11). However, we present a new derivation that will be useful to generalize it into a demic-cultural model in the next subsection.

For human populations, newborn children cannot survive on their own. However, when they come on age they can move away from their parents. As explained below, this point has led some authors to use equations of the so-called cohabitation type (9-11). Let us consider the following one

$$N(x,y,t+T) = \int_{-\infty}^{\infty}\int_{-\infty}^{\infty} r\left[N\left(x+\Delta_x, y+\Delta_y, t\right)\right]\ \phi_N^{children}\left(\Delta_x,\Delta_y\right) d\Delta_x\, d\Delta_y + \int_{-\infty}^{\infty}\int_{-\infty}^{\infty} s\left[N\left(x+\Delta_x, y+\Delta_y, t\right)\right]\ \phi_N^{adults}\left(\Delta_x,\Delta_y\right) d\Delta_x\, d\Delta_y, \qquad \textbf{[S1]}$$

where $N(x,y,t)$ is the population density (of farmers in our case) at position (x,y) and time t. T is the generation time, here defined as the mean age difference between a parent and her/his children. The reproduction function $r[N]$ is the number of children (per unit area) who are born and do not die during the time interval T, if the initial population density is N. Similarly, the survival function $s[N]$ is the number of initial individuals (also per unit area) who survive during the time interval T. The dispersal kernels $\phi_N^i(\Delta_x,\Delta_y)$ are defined as the probability to move between $(x+\Delta_x, y+\Delta_x)$ and $(x+\Delta_x, y+\Delta_x)$ during the time interval T. Equation **S1** includes the effect of dispersal of the new generation (first term on the right) as well as that of the old generation (last term), because individuals of both generations can change their place of residence in general. For simplicity we assume a single dispersal kernel $\phi_N(\Delta_x,\Delta_y)$ for both generations of farmers (this is reasonable according to ethnographic data (12)). Then Eq. **S1** simplifies into

$$N(x,y,t+T) = \int_{-\infty}^{\infty}\int_{-\infty}^{\infty} R_T\left[N\left(x+\Delta_x, y+\Delta_y, t\right)\right]\ \phi_N\left(\Delta_x,\Delta_y\right) d\Delta_x\, d\Delta_y, \qquad \textbf{[S2]}$$

where

$$R_T[N] = r[N] + s[N]. \qquad \textbf{[S3]}$$

According to ethnographic data (see (13) and references therein), the joint effects of reproduction and survival are well-described by a logistic growth function, namely $R_T[N(x,y,t)] = \frac{e^{a_N T}\, K_N\, N(x,y,t)}{K_N + \left(e^{a_N T}-1\right) N(x,y,t)}$. Then the speed of front solutions to Eq. **S2** is given by Eq. **3** in the main paper (9-11).

Clearly the cohabitation Eq. **S2** is more reasonable than the following, non-cohabitation equation for the change in population density (13)

$$N(x,y,t+T) - N(x,y,t) = \int_{-\infty}^{\infty}\int_{-\infty}^{\infty} N\left(x+\Delta_x, y+\Delta_y, t\right)\ \phi_N\left(\Delta_x,\Delta_y\right) d\Delta_x\, d\Delta_y - N(x,y,t) + R_T[N(x,y,t)] - N(x,y,t), \qquad \textbf{[S4]}$$

which, using Eq. **S3**, can be written as

$$N(x,y,t+T)=\int_{-\infty}^{\infty}\int_{-\infty}^{\infty}N\left(x+\Delta_x,y+\Delta_y,t\right)\ \phi_N\left(\Delta_x,\Delta_y\right)d\Delta_x\,d\Delta_y-N(x,y,t)+$$
$$r[N(x,y,t)]+s[N(x,y,t)]. \qquad \textbf{[S5]}$$

The reason why Eq. **S2** is more reasonable than Eq. **S4** or **S5** is that, clearly, Eq. **S5** assumes that individuals born at (x,y) at time t (last-but-one term) will not move at all, i.e. they will all still be at (x,y) on coming of age (time $t+T$, left-hand side). Thus, for example, in the simple case in which all parents move, they will leave all of their children alone. Such an anthropologically unrealistic feature makes it clear that Eq. **S5** is less accurate than Eq. **S2**. For additional derivations and figures showing that Eq. **S4** is less realistic that the cohabitation Eq. **S2**, see especially Fig. 1 of Ref. (10), Fig. 17 of Ref. (14), and Ref. (9).

Fisher's model (Eqs. **1-2** in the main paper) is obtained from the less accurate, non-cohabitation model (Eq. **S4**) by performing Taylor expansions and assuming an isotropic kernel (see below, section *Ordinary diffusion*). Thus Fisher's model is clearly less realistic than the cohabitation model (Eq. **S2**). A second way to see the limitations of Fisher's speed (Eq. **2** in the main paper), namely $s_F=2\sqrt{a_N D_N}$, yields $s_F\to\infty$ for $a_N\to\infty$ and, in contrast, numerical simulations have shown that the cohabitation-kernel model (Eq. **3** in the main paper) yields for $a_N\to\infty$ the value $s=r_{max}/T$, i.e. the maximum dispersal distance divided by the generation time (15,11), which is physically reasonable. We thus prefer to use Eq. **3** rather than Fisher's speed (Eq. **2**). A third reason for this is that, in purely-demic models, the error of Fisher's Eq. **2** relative to Eq. **3** reaches 30% for realistic kernels and parameter values (see below, section *Ordinary diffusion*). Thus we use cohabitation-kernel equations (i.e., of the type of Eq. **S2**) rather than ordinary diffusion equations (i.e., of the type of Fisher's Eq. **1**). The fourth reason to use cohabitation-kernel equations is that, when cultural transmission is included (next paragraphs), Fisher-type Eqs. lead to very large errors (see below, section *Ordinary diffusion*).

Demic-cultural model. When the conversion of hunter-gatherers into farmers (cultural transmission) is taken into account, we might be tempted to generalize Eq. **S2** into

$$N(x,y,t+T)=\int_{-\infty}^{\infty}\int_{-\infty}^{\infty}R_T\left[N\left(x+\Delta_x,y+\Delta_y,t\right)\right]\ \phi_N\left(\Delta_x,\Delta_y\right)d\Delta_x\,d\Delta_y+$$
$$\int_{-\infty}^{\infty}\int_{-\infty}^{\infty}c\left[N\left(x+\Delta_x,y+\Delta_y,t\right),P\left(x+\Delta_x,y+\Delta_y,t\right)\right]\ \phi_N^{converts}\left(\Delta_x,\Delta_y\right)d\Delta_x\,d\Delta_y \qquad \textbf{[S6]}$$

and a similar equation for the population density of hunter-gatherers P (with a minus sign in the last term). The cultural transmission function $c[\ldots]$ in Eq. **S6** is due to the conversion of hunter-gatherers into farmers (and is similar to the reproduction $r[\ldots]$ and survival $s[\ldots]$ functions in Eq. **S1**). Recently its has been shown that this cultural transmission function is given by (see Eq. **1** in Ref. (11) and its derivation therein)

$$c[N(x,y,t),P(x,y,t)]=f\,\frac{N(x,y,t)\,P(x,y,t)}{N(x,y,t)+\gamma\,P(x,y,t)}, \qquad \textbf{[S7]}$$

where f and γ are cultural transmission parameters. The kernel $\phi_N^{converts}(\Delta_x,\Delta_y)$ in Eq. **S6** is the dispersal kernel of hunter-gatherers that have been converted into farmers. Since they now

behave as farmers, it is reasonable to assume that this kernel is the same as the kernels $\phi_N^{children}(\Delta_x,\Delta_y)$ and $\phi_N^{adults}(\Delta_x,\Delta_y)$ in Eq. **S1**. Then Eq. **S6** becomes

$$N(x,y,t+T)=\int_{-\infty}^{\infty}\int_{-\infty}^{\infty} R_T[N(x+\Delta_x,y+\Delta_y,t)]\ \phi_N(\Delta_x,\Delta_y)\,d\Delta_x\,d\Delta_y + \int_{-\infty}^{\infty}\int_{-\infty}^{\infty} f\,\frac{N(x+\Delta_x,y+\Delta_y,t)\,P(x+\Delta_x,y+\Delta_y,t)}{N(x+\Delta_x,y+\Delta_y,t)+\gamma\,P(x+\Delta_x,y+\Delta_y,t)}\ \phi_N(\Delta_x,\Delta_y)\,d\Delta_x\,d\Delta_y \quad \textbf{[S8]}$$

A model of this kind was applied recently (see Eq. **5** in Ref. (11)). It is an approximation that may be valid in some regions (with mainly demic diffusion) but it cannot lead to a purely cultural model of Neolithic spread (because according to Eq. **S8** there is no front propagation in the absence of demic diffusion, since then $\phi_N(\Delta_x,\Delta_y)\neq 0$ only at vanishing distance, i.e. $\Delta=(\Delta_x^2+\Delta_y^2)^{1/2}=0$). Thus we will here consider a more realistic model in two ways. Firstly we take into account that, according to ethnographic observations, hunter-gatherers can learn agriculture from farmers located some distance away (empirical data and references are given below, section *Speed ranges predicted by the models).* Then Eq. **S8** is generalized into

$$N(x,y,t+T)=\int_{-\infty}^{\infty}\int_{-\infty}^{\infty} R_T[N(x+\Delta_x,y+\Delta_y,t)]\ \phi_N(\Delta_x,\Delta_y)\,d\Delta_x\,d\Delta_y + \int_{-\infty}^{\infty}\int_{-\infty}^{\infty}\phi_N(\Delta_x,\Delta_y)\,d\Delta_x\,d\Delta_y \int_{-\infty}^{\infty}\int_{-\infty}^{\infty}\phi'_P(\Delta'_x,\Delta'_y)\,d\Delta'_x\,d\Delta'_y\ f\,\frac{N(x+\Delta_x+\Delta'_x,y+\Delta_y+\Delta'_y,t)\,P(x+\Delta_x,y+\Delta_y,t)}{N(x+\Delta_x+\Delta'_x,y+\Delta_y+\Delta'_y,t)+\gamma\,P(x+\Delta_x,y+\Delta_y,t)}, \quad \textbf{[S9]}$$

where in practice, the cultural kernel $\phi'_P(\Delta'_x,\Delta'_y)$ is a set of probabilities P_k for hunter-gatherers to learn agriculture from farmers living at distances $R_k=(\Delta'^2_x+\Delta'^2_y)^{1/2}$, during a generation time T. Similarly, in practice the demic kernel $\phi_N(\Delta_x,\Delta_y)$ in Eqs. **S2-S8** is a set of probabilities p_j for farmers to disperse at distances $r_j=(\Delta_x^2+\Delta_y^2)^{1/2}$, also during a generation time T (9-11).

Secondly we note that in Eq. **S1** we have taken into account that after a generation time T, only part of the initial population will be alive (the survival function $s[\ldots]$ in the last term) and that reproduction will have lead to new individuals (reproduction function $r[\ldots]$ in the last term). Obviously both processes will in general have an effect also on the population of converts (last term in Eq. **S9**). Thus we finally generalize Eq. **S9** into

$$N(x,y,t+T)=\int_{-\infty}^{\infty}\int_{-\infty}^{\infty} R_T[N(x+\Delta_x,y+\Delta_y,t)]\ \phi_N(\Delta_x,\Delta_y)\,d\Delta_x\,d\Delta_y + \int_{-\infty}^{\infty}\int_{-\infty}^{\infty}\phi_N(\Delta_x,\Delta_y)\,d\Delta_x\,d\Delta_y \int_{-\infty}^{\infty}\int_{-\infty}^{\infty}\phi'_P(\Delta'_x,\Delta'_y)\,d\Delta'_x\,d\Delta'_y\ R_T\left[f\,\frac{N(x+\Delta_x+\Delta'_x,y+\Delta_y+\Delta'_y,t)\,P(x+\Delta_x,y+\Delta_y,t)}{N(x+\Delta_x+\Delta'_x,y+\Delta_y+\Delta'_y,t)+\gamma\,P(x+\Delta_x,y+\Delta_y,t)}\right], \quad \textbf{[S10]}$$

with $R_T[N]=r[N]+s[N]$ (see Eq. **S3**). It is easy to show, by following exactly the same steps as in the *Methods* section of Ref. (11), that the speed of front solutions to Eq. **S10** is given by Eq. **4** in the main paper, with $C=f/\gamma$. This reduced parameter C was called the intensity of cultural transmission (11) because, according to Eq. **S7**, $C=f/\gamma$ is the number of hunter-gatherers converted per farmer at the front leading edge (i.e. in regions such that $N\ll P$) (11). Without cultural transmission ($C=0$), the demic-cultural front speed (Eq. **4** in the main paper) reduces to the purely-demic speed (Eq. **3** in the main paper), as it should. With frequency-dependent cultural transmission, the equations are longer but the final results are exactly the same (see below, section *Frequency-dependent cultural transmission*).

It is important to note that cultural transmission (the factor in brackets $[f \dots]$ at the end of the second line in Eq. [S10]) is applied in a term that also contains the effects of net reproduction (R_T) and dispersal (the kernel of farmers $\phi_N(\Delta_x, \Delta_y)$). Thus some hunter-gatherers will learn agriculture from farmers located a distance (Δ'_x, Δ'_y), and the children of those converted hunter-gatherers will possibly move a distance (Δ_x, Δ_y) (similarly to the children of farmers, first line). Therefore, some hunter-gatherers can learn agriculture from farmers and the next generation (i.e., the children) of those hunter-gatherers will be farmers. Such a conversion during a generation time is reported by ethnographic data (16) and implies that the individual acculturation process is not instantaneous but takes place within one generation time, which seems reasonable for a complex cultural trait as farming.

Purely cultural model. This model corresponds to considering that the demic kernel $\phi_N(\Delta_x, \Delta_y)$ is different from zero only at at $\Delta_x \approx 0$, $\Delta_y \approx 0$, so that we can replace the corresponding integral over all distances in Eq. **S10** by the integrand evaluated at $\Delta_x \approx 0$, $\Delta_y \approx 0$. Then

$$N(x,y,t+T) = R_T[N(x,y,t)] + \int_{-\infty}^{\infty}\int_{-\infty}^{\infty} \phi'_P(\Delta'_x, \Delta'_y)\, d\Delta'_x\, d\Delta'_y\ R_T\left[f\, \frac{N(x+\Delta'_x, y+\Delta'_y, t)\, P(x,y,t)}{N(x+\Delta'_x, y+\Delta'_y, t) + \gamma\, P(x,y,t)}\right] \quad \textbf{[S11]}$$

and applying again the steps in the *Methods* section of Ref. (11), we find that the speed of purely cultural fronts is given by Eq. **5** in the main paper. Alternatively, the purely cultural speed (Eq. **5**) can be also obtained as a special case of the demic-cultural speed (Eq. **4**) by considering the case without demic diffusion (i.e., $r_1 = 0$ and $p_1 = 1$) and taking into account that the definition $I_0(\lambda r_j) = \frac{1}{2\pi}\int_0^{2\pi} d\theta\, \exp[-\lambda r_j \cos\theta]$ implies that $I_0(0) = 1$.

Note that the purely cultural model (Eq. **S11**) neglects the effect of the dispersal of children whose parents practice farming. Thus in this approximation farming spreads only because it is taught by converted hunter-gatherers (with population density $N(x,y,t)$) to non-converted hunter-gatherers (with population density $P(x,y,t)$) living some distance away. The dispersal of children is not taken into account, neither for farmers (because it is assumed that they do not disperse into the region populated by hunter-gatherers, in contrast to the demic-cultural model above) nor for converted hunter-gatherers. Below we generalize this purely cultural model by a more complicated one that includes a short-range dispersal kernel for converted hunter-gatherers (section *Effect of a short-range dispersal kernel*).

S3. Speed ranges predicted by the models. ***Speed range from the purely demic model.*** We consider the ranges for a_N and T that have been measured for preindustrial farming populations, namely $0.023\, y^{-1} \leq a_N \leq 0.033\, y^{-1}$ and $29 \leq T \leq 35\, y$ (see the *SI Appendix* to Ref. (11) for details). We also apply the 5 dispersal kernels $\phi_N(\Delta_x, \Delta_y)$ (i.e., sets of probabilities p_j versus distances r_j) given below, which were measured for preindustrial farming populations (10). Obviously, for each kernel the slowest speed is obtained for the lowest observed value of the initial reproduction rate of farmers ($a_N = 0.023\, y^{-1}$) and the highest observed value of the generation or mean delay time between the dispersal of parents and their children (17) ($T =$

35 y). The fastest speed is obtained for $a_N = 0.033\ y^{-1}$ and $T = 29\ y$. Using these values and each kernel in Eq. **3** yields the following purely-demic speed ranges,

Population A (Gilishi 15) in Ref. (10): demic kernel $\{p_j\}$={0.54, 0.17, 0.04, 0.25}, $\{r_j\}$={2.4; 14.5, 36.3, 60.4}km, speed range 0.87-1.15 km/y.

Population B (Gilishi 25) in Ref. (10): demic kernel $\{p_j\}$={0.40, 0.17, 0.17, 0.26}, $\{r_j\}$={2.4; 14.5, 36.3, 60.4}km, speed range 0.92-1.21 km/y.

Population C (Shiri 15) in Ref. (10): demic kernel $\{p_j\}$={0.19, 0.07, 0.22, 0.52}, $\{r_j\}$={2.4; 14.5, 36.2, 60.4}km, speed range 1.14-1.48 km/y.

Population D (Yanomamö) in Ref. (10): demic kernel $\{p_j\}$={0.19, 0.54, 0.17, 0.04, 0.04, 0.02}, $\{r_j\}$={5, 30, 50, 70, 90, 110}km, speed range 1.12-1.48 km/y.

Population E (Issongos) in Ref. (10): demic kernel $\{p_j\}$={0.42; 0.23; 0.16; 0.08; 0.07; 0.02; 0.01; 0.01}, $\{r_j\}$={2.3, 7.3, 15, 25, 35, 45, 55, 100}km, speed range 0.68-0.92 km/y.

The overall range predicted by the purely demic model is thus $0.68 < s_D < 1.48$ km/y. This is the range applied in the main papers, *Results.*

The 5 dispersal kernels above have been measured for pre-industrial populations. It is very difficult to find such kind of data. There are some 19th-20th century kernels for industrialized regions in, e.g., Europe (18), but there was a dramatic increase in dispersal distances with the arrival of mechanized forms of transport in the middle of the 19th century (19) so it does not seem possible to apply such kernels to the Neolithic transition.

It is reasonable to ask to what extent the 5 pre-industrial dispersal kernels above are representative of the wide range of geographic regions in Europe. For example, we might perhaps expect to observe narrower kernels in mountainous regions. However this does not seem to be the case because, by comparing several pre-industrial populations, some authors have observed an increase of dispersal distance in areas with lower population densities (20-23), as is usually the case for mountainous regions. Unfortunately, for most populations only mean dispersal distances have been reported (20-23) but the complete probability distribution (kernel) is necessary to realistically predict demic speeds (because the kernel introduces corrections of up to 50% as compared to mean distances (10)). We thus focus our attention on the geographic features of the 5 pre-industrial populations above (for which dispersal kernels have been reported, rather than only mean dispersal distances).

Populations A (Gilishi 15), B (Gilishi 25) and C (Shiri 15): these are 3 groups of the Majangir, who are pre-industrial agriculturalists in Ethiopia. Stauder conducted fieldwork with them during 2 years and reported dispersal kernels as histograms of the number of people for each distance class (see Ref. (12), p. 139, which includes additional groups that are not representative according to Stauder because they were subject to raids by foreign tribes, as also discussed in Ref. (24), pp. 80 and 155). The Majangir live at all altitudes in the range 600-1,500 m above sea level. The climate is characterized by high humidity and abundant precipitation (12). As seen above, the speed range implied by these kernels is 0.87-1.48 km/y.

Population D (Yanomamö): these pre-industrial horticulturalists live in Southern Venezuela and the adjacent portions of Northen Brazil. A dispersal kernel was published by MacDonald and Hewlett (20), based on data supplied by N. A. Chagnon. The groups where Chagnon conducted fieldwork live at altitudes in the range 100-400 m, in a flat plain interrupted occasionally by gently rolling hills where there is no lack of water, specially during the wet season (when most of the agricultural tasks are performed) (25). As seen above, the speed range implied by this kernel is 1.12-1.48 km/y. Therefore, this population lives at lower altitudes but has similar demic speeds to populations A-C above. This confirms that lower altitudes do not necessarily lead to narrower kernels and slower demic speeds.

Population E (Issongos): these pre-industrial cultivators live in the Lobaye forest in the Central African Republic, at altitudes in the range 450-700 m. Interestingly, they live side by side with Aka pygmies, who are hunter-gatherers (see below). In the rain forest, herbs are less common than in other environments, thus less care is needed and cultivators can abandon their fields for long periods (26). A dispersal kernel for the Issongos was published by Cavalli-Sforza and Bodmer (Ref. (27), Fig. 8.16.B). As seen above, it implies the speed range 0.68-0.92 km/y. This is slower than the ranges for the 4 populations above (because almost no individuals disperse further away than 35 km for population E, whereas a substantial portion does for populations A-D). Thus we note that the demic speed of population E is slower, although it lives at intermediate altitudes. Therefore, no relationship between altitude and demic speed is implied by the data.

The descriptions above imply a very wide range of altitudes and geographic environments for populations A-E. Therefore, in our opinion these data can be representative of the wide range of altitudes and geographical features in Europe.

Let us recall that in the main paper we have found that the minimum demic speed is 0.68 km/y, and that this is rather similar to the maximum cultural speed (0.66 km/y). This minimum demic speed (0.68 km/y) corresponds to population E, which lives at intermediate altitudes but, for some reason, has a substantially narrower kernel than the other 4 populations. If population E had been considered an outlier and excluded from the analysis, the minimum demic speed would have been 0.87 km/yr, i.e. substantially faster than the maximum cultural speed (0.66 km/y). However, we have preferred to use all pre-industrial kernels available, mainly because all of them come from ethnographic studies, due to the fact that unfortunately it has not been yet possible to measure dispersal kernels for prehistoric populations. This confirms the need of such studies (a possible procedure based on Genetics is proposed in the main paper, *Discussion*).

Speed range from the purely cultural model. The cultural transmission intensity C from hunter-gathering to farming was estimated from several case studies in Ref. (11) and the overall range is $1.0 \leq C \leq 10.9$. In those case studies, cultural transmission was local because the hunter-gatherers live in the same place as the farmers. But hunter-gatherers also learn agricultural techniques from nearby farmers (28). This corresponds to the last term in Eq. **S9**, where the cultural kernel $\phi_P'(\Delta_x, \Delta_y)$ is a set of probabilities P_k for hunter-gatherers to learn agriculture

from farmers living at distances R_k. We estimated 5 cultural kernels from populations of hunter-gatherers that practice agriculture as follows. Hart (29,30) observed Mbuti pygmies during about 1 year and noted that they performed agricultural work and exchanged products with several villages of farmers simultaneously (not only with the closest village). A map by Hart (30) makes it possible to measure the distances from the hunter-gatherers camp locations (which changed every four weeks on average) to the villages of farmers. This yields the following cultural kernels. For each kernel, we also report the purely-cultural speed range obtained from Eq. **5** with $a_N = 0.023\ y^{-1}$, $T = 35\ y$ and $C = 1$ (slowest speed) or $a_N = 0.033\ y^{-1}$, $T = 29\ y$ and $C = 10.9$ (fastest speed).

Population 1 (Mbuti, band I in Ref. (30)): cultural kernel $\{P_k\}$={0.59, 0.37, 0.04}, $\{R_k\}$={2.5, 7.5, 12.5}km, speed range 0.17-0.36 km/y.

Population 2 (Mbuti, band II in Ref. (30)): cultural kernel $\{P_k\}$={0.12, 0.30, 0.43, 0.15}, $\{R_k\}$={2.5, 7.5, 12.5, 17.5}km, speed range 0.30-0.57 km/y.

Population 3 (Mbuti, band III in Ref. (30)): cultural kernel $\{P_k\}$={0.20, 0.41, 0.26, 0.08, 0.05}, $\{R_k\}$={2.5, 7.5, 12.5, 17.5, 22.5}km, speed range 0.32-0.66 km/y.

The following distances are perhaps less precise (because they refer to the nearest farmer village, not necessarily to all villages with cultural transmission to hunter-gatherers).

Population 4 (Aka (31)): cultural kernel $\{P_k\}$={0.12, 0.25, 0.11, 0.04, 0.03, 0.16, 0.05, 0.05, 0.05, 0.14}, $\{R_k\}$={0.05, 0.1, 0.2, 0.25, 0.3, 0.4, 2, 3, 5, 6}km, speed range 0.09-0.19 km/y.

Population 5 (Baka (32)): cultural kernel $\{P_k\}$={0.48, 0.04, 0.13, 0.14, 0.18, 0.03}, $\{R_k\}$={0, 0.5, 0.8, 1.5, 1.7, 2.7}km, speed range 0.03-0.07 km/y.

Thus the purely cultural model yields 0.03-0.66 km/y. This is the range applied in the main papers, *Results*.

It is worth to note that the maximum distances of the 5 cultural kernels (populations 1-5) are substantially smaller than the maximum distances of the 5 demic kernels (populations A-E). This seems to indicate that agriculture cannot be culturally transmitted at long distances, presumably because it is a complex cultural trait to learn (in contrast, simpler cultural traits such as new ceremonies, ochre and shells are transmitted by hunter-gatherers at distances of up to several hundred kilometers (33)).

Finally we briefly discuss the geographical environment of populations 1-5, for which we have used the cultural kernels (similarly to the descriptions above of populations A-E, for which we have used the demic kernels).

Populations 1-3 (Mbuti): these hunter-gatherers live in the Ituri forest (Democratic Republic of Congo, former Zaire), which has an altitude range of 700-1,000m. As mentioned above, each group of them performs agricultural work for several villages of farmers (who live in small clearings of the forest) in exchange for cultivated food and other items. The availability of forest foods is closely tied to rainfall, which is generally high (but lower in winter) (29,30). The cultural speed range for these populations is 0.17-0.66 km/y (see above).

Population 4 (Aka): these hunter-gatherers live in the Lobaye forest (Central African Republic), which has an altitude range of 450-700m (see the description of the Issongo farmers above). The less acculturated ones (who have not yet adopted the language of farmers) usually go away to hunt deep in the forest most of the year and return to live near the farmers' villages in the winter (dry season), when they work for the farmers (31). The cultural speed range is 0.09-0.19 km/y (see above).

Population 5 (Baka): these hunter-gatherers live in the Sangha region of northwestern Congo, which has an altitude range of 300-600m. These hunter-gatherers normally conduct one-day hunting trips, and occasionally go away to hunt for 2 or 3 weeks (32). The cultural speed range is 0.03-0.07 km/y (see above).

According to Sato (32), Baka-farmer relationships are considerably different from Mbuti-famer or Aka-farmer relationships because there are many more farmers than Baka hunter-gatherers, so the latter have an advantage over the former in the negotiation to exchange work for food and other items. Thus our data include a variety of cultural interactions, as well as altitudes and geographic environments.

We note that for populations 4 and 5, the cultural speeds are substantially slower than for populations 1-3. As mentioned above, the cultural kernels for populations 4-5 may be less precise because only distances to the nearest farmer village were recorded (not to all villages with cultural transmission). This difference explains why their cultural transmission kernels are narrower, and therefore the cultural speeds slower. In view of this, although it is true that populations 4-5 have both slower cultural speeds and lower altitudes, we suspect that altitude is not an important factor in determining cultural speeds. If additional cultural kernels are obtained in the future, it will be possible to perform more estimations. However, at this point neglecting populations 4 and 5 would not change the result in the main paper that the maximum cultural speed is 0.66 km/y (rather similar to the minimum demic speed, 0.68 km/y), because the value 0.66 km/y corresponds to population 3. Moreover, the maximum speed of populations 1 and 2 are 0.36 km/y and 0.57 km/y, rather similar to that for population 3 (0.66 km/y) compared to those for populations 4 (0.19 km/y) and 5 (0.07 km/y).

Speed range from the demic-cultural model. The slowest speed predicted by the demic-cultural model obviously corresponds to neglecting cultural transmission ($C = 0$, i.e. to the purely demic model), the slowest dispersal kernel (population E), the lowest observed value of a_N ($0.023\ y^{-1}$) and the highest observed value of T ($35\ y$), namely 0.68 km/y (see the purely demic model above).

The relevant result of the demic-cultural model is its fastest speed. This obviously corresponds to the strongest observed value for the intensity of cultural transmission ($C = 10.9$), the fastest cultural kernel (population 3), the fastest demic kernel (population C or D), the highest observed value of a_N ($0.033\ y^{-1}$) and the lowest observed value of T ($29\ y$). Using these data into Eq. **4** we find that the fastest speed is obtained for the demic kernel of population D and is 3.04 km/y. This is the value applied in the main papers, *Results.*

S4. Frequency-dependent cultural transmission. In *Methods* we have considered the simplest possible case of cultural transmission (namely, a frequency-independent value of f) to avoid using rather long equations. However, in many examples of cultural transmission f depends on frequency (this effect is often called conformist transmission) (34-36). Here we show that in the frequency-dependent case, all of the results in our paper are exactly the same. To see this, we recall that in the frequency-independent case the cultural transmission equations in homogeneous systems are, according to Eq. **S7** (see also Eqs. **1** in Ref. (11) and the derivation there),

$$\begin{cases} P_N\ (t+T) = P_N(t) + f\frac{P_N(t)\, P_P(t)}{P_N(t)+\gamma P_P(t)} \\ P_P\ (t+T) = P_P(t) - f\frac{P_N(t)\, P_P(t)}{P_N(t)+\gamma P_P(t)}, \end{cases} \quad \textbf{[S12]}$$

where P_N is the number of farmers, P_P the number of hunter-gatherers, t is the time, $T = 1$ generation, and f and γ are cultural transmission parameters (11).

In the frequency-dependent case, Eqs. **S12** are generalized into (see Eqs. **S3** in Ref. (11))

$$\begin{cases} P_N\ (t+T) = P_N(t) + \frac{P_N(t)\, P_P(t)}{P_N(t)+\gamma P_P(t)}\left(f + h\left[2\frac{P_N(t)}{P_N(t)+P_P(t)} - 1\right]\right) \\ P_P\ (t+T) = P_P(t) - \frac{P_N(t)\, P_P(t)}{P_N(t)+\gamma P_P(t)}\left(f + h\left[2\frac{P_N(t)}{P_N(t)+P_P(t)} - 1\right]\right), \end{cases} \quad \textbf{[S13]}$$

and the frequency-independent case (Eqs. **S12**) is recovered for $h = 0$. Therefore, in the frequency-dependent case, in the last term in Eq. **S10** (demic-cultural model) $f\frac{N(x+\Delta_x+\Delta'_x,y+\Delta_y+\Delta'_y,t)\, P(x+\Delta_x,y+\Delta_y,t)}{N(x+\Delta_x+\Delta'_x,y+\Delta_y+\Delta'_y,t)+\gamma\, P(x+\Delta_x,y+\Delta_y,t)}$ is replaced by

$$\frac{N(x+\Delta_x+\Delta'_x,y+\Delta_y+\Delta'_y,t)\, P(x+\Delta_x,y+\Delta_y,t)}{N(x+\Delta_x+\Delta'_x,y+\Delta_y+\Delta'_y,t)+\gamma\, P(x+\Delta_x,y+\Delta_y,t)}\left(f + h\left[2\frac{N(x+\Delta_x+\Delta'_x,y+\Delta_y+\Delta'_y,t)}{N(x+\Delta_x+\Delta'_x,y+\Delta_y+\Delta'_y,t)+P(x+\Delta_x,y+\Delta_y,t)} - 1\right]\right). \quad \textbf{[S14]}$$

Analogously, in the last term in Eq. **S11** (purely cultural model) $f\frac{N(x+\Delta'_x,y+\Delta'_y,t)\, P(x,y,t)}{N(x+\Delta'_x,y+\Delta'_y,t)+\gamma\, P(x,y,t)}$ is replaced by

$$\frac{N(x+\Delta'_x,y+\Delta'_y,t)\, P(x,y,t)}{N(x+\Delta'_x,y+\Delta'_y,t)+\gamma\, P(x,y,t)}\left(f + h\left[2\frac{N(x+\Delta'_x,y+\Delta'_y,t)}{N(x+\Delta'_x,y+\Delta'_y,t)+P(x,y,t)} - 1\right]\right). \quad \textbf{[S15]}$$

As shown in detail in the *Methods* section in (11), the front speed is found by considering the leading edge of the front, where $N(\ldots) << P(\ldots)$ and linearizing. Then

$$\frac{N(\ldots)\, P(\ldots)}{N(\ldots)+\gamma\, P(\ldots)}\left(f + h\left[2\frac{N(\ldots)}{N(\ldots)+\gamma\, P(\ldots)} - 1\right]\right) \approx \frac{1}{\gamma}(f-h)\, N(\ldots) + O(N^2), \quad \textbf{[S16]}$$

where $C \equiv f/\gamma$ and $O(N^2)$ are higher-order terms. In this way, and repeating exactly the same steps as in the *Methods* section in (11), it is easily found that the speeds of the demic-cultural and purely cultural models (Eqs. **4** and **5** in the main paper, respectively) in the frequency-dependent case ($h \neq 0$) are the same as in the frequency-independent case ($h = 0$) with $C \equiv f/\gamma$ replaced by $C - h/\gamma$. Therefore, the equations for the speed in the frequency-independent case ($h = 0$) are different than in the frequency-dependent case ($h \neq 0$). However, in the frequency-independent case the observed range of C was estimated by noting that if $P_N << P_P$ the first Eq. **S12** becomes $P'_N = (1 + C)P_N$ (see *SI Appendix* in (11)). But in the frequency-dependent case, if $P_N << P_P$ the first Eq. **S12** becomes $P'_N = (1 + C - h/\gamma)P_N$. Thus we conclude that the case studies that yield the observed range $1.0 \leq C \leq 10.9$ in the frequency-independent case (see *SI Appendix* in (11)) will surely yield the observed range $1.0 \leq C - h/\gamma \leq 10.9$ in the frequency-dependent case. Therefore, although the equations are different, the speed ranges predicted by the models will be exactly the same for the frequency-independent case (main paper) than for the more general, frequency-dependent case.

S5. Ordinary diffusion. Here we compare Fisher's equation with cultural transmission to the more accurate model in the main paper (see section *Models* above). We will find that both approaches yield a similar speed range for the purely demic case, but ordinary diffusion leads to very large errors when cultural transmission is included.

We first include the effect of cultural transmission to a previous derivation of Fisher's Eq. **1** (13). The non-cohabitation Eq. **S4** is purely demic, but we can include cultural transmission (see the last term in Eq. **S11**),

$$N(x,y,t+T) - N(x,y,t) = \int_{-\infty}^{\infty}\int_{-\infty}^{\infty} N(x+\Delta_x, y+\Delta_y, t)\ \phi_N\left(\Delta_x, \Delta_y\right) d\Delta_x\, d\Delta_y - N(x,y,t) +$$

$$R_T[N(x,y,t)] - N(x,y,t) + \int_{-\infty}^{\infty}\int_{-\infty}^{\infty} \phi_P^{'}\left(\Delta'_x, \Delta'_y\right) d\Delta'_x\, d\Delta'_y\ f\,\frac{N\left(x+\Delta_x^{'}, y+\Delta_y^{'}, t\right) P(x,y,t)}{N\left(x+\Delta_x^{'}, y+\Delta_y^{'}, t\right) + \gamma\, P(x,y,t)} \quad \textbf{[S17]}$$

(and an analogous equation holds for the hunter-gatherer population density P, with a minus sign for the last term). Recall that $\phi_P'\left(\Delta'_x, \Delta'_y\right)$ is the cultural kernel, so the last term in Eq. **S17** includes only the effect of cultural transmission (not of demic dispersal neither reproduction). Similarly the first and second terms on the right-hand side include only the effect of demic dispersal, and the third and fourth terms include only reproduction (this additive property is called non-cohabitation and is necessary to recover Fisher's equation, see the next paragraph). Assuming isotropic kernels and performing Taylor expansions up to second order in space and first order in time, we obtain that

$$\begin{cases} \dfrac{\partial N}{\partial t} = D_N \nabla^2 N + F(N) + \dfrac{f}{T}\,\dfrac{N\,P}{N+\gamma\,P} + f\,\dfrac{P}{N+\gamma\,P}\,D'\,\nabla^2 N \\ \dfrac{\partial P}{\partial t} = D_P \nabla^2 P + F(P) - \dfrac{f}{T}\,\dfrac{N\,P}{N+\gamma\,P} - f\,\dfrac{P}{N+\gamma\,P}\,D'\,\nabla^2 N, \end{cases} \quad \textbf{[S18]}$$

where $D_N = \frac{1}{4T}\sum_{j=1}^{M} p_j r_j^2$ is the usual, *demic* diffusion coefficient (13) of the Neolithic population (farmers) and D_P is the analogous *demic* diffusion coefficient for hunter-gatherers. The new parameter $D' = \frac{1}{4T}\sum_{k=1}^{N} P_k R_k^2$ can be similarly called the *cultural* diffusion coefficient. As in Refs. (11) and (13), $F(N) = a_N N\left(1 - \frac{N}{K_N}\right)$ and $F(N) = a_N N\left(1 - \frac{N}{K_N}\right)$ are logistic growth terms (with a_i the initial growth rate and K_i the carrying capacity of population $i = N, P$). For simplicity we have omitted higher-order terms (namely, a term with $N\,\nabla^2 N$ and a term with $(\nabla N)^2$) because they have not effect on the front speed (this is easily seen by using the linearization procedure explained in the Methods section of Ref. (11)).

In the absence of cultural transmission ($f = 0$), Eq. **S18** reduces to Fisher's model (Eq. **1** in the main paper**)**. If cultural transmission is local ($D' = 0$), Eq. **S18** reduces to a previous model that did not take into account that hunter-gatherers can learn agriculture from farmers living at other places (Eq. **S10** in Ref. (11)).

By using the linearization procedure explained in the Methods section of Ref. (11), it is found that the speed of the waves of advance of farmers for the ordinary-diffusion model described by equations **S18** is

$$s_{diff} = 2\sqrt{\left(a_N + \frac{C}{T}\right)(D_N + C\,D')}, \qquad \textbf{[S19]}$$

where $C = f/\gamma$. In the absence of cultural transmission ($C = 0$) Eq. **S19** reduces to Fisher's speed (Eq. **2** in the main paper), namely $s_F = 2\sqrt{a_N D_N}$ (37). If there is cultural transmission but only locally (i.e. without non-local cultural transmission, $D' = 0$) Eq. **S19** reduces to Eq. **S11** in Ref. (11).

Using Eq. **S19** and the same parameter ranges as above (section *Speed ranges predicted by the models*), we find the following results.

Purely demic model with ordinary diffusion ($C = 0$ in Eq. **S19**, Fisher's speed $s_F = 2\sqrt{a_N D_N}$ (37)). For each population, again the slowest speed is obtained for $a_N = 0.023\ y^{-1}$ and $T = 35\ y$, and the fastest one for $a_N = 0.033\ y^{-1}$ and $T = 29\ y$.

Population A (Gilishi 15) in Ref. (10): $D_N = 251$ km$^2/T$, speed range 0.81-1.07 km/y.

Population B (Gilishi 25) in Ref. (10): $D_N = 302$ km$^2/T$, speed range 0.89-1.17 km/y.

Population C (Shiri 15) in Ref. (10): $D_N = 550$ km$^2/T$, speed range 1.20-1.58 km/y.

Population D (Yanomamö) in Ref. (10): $D_N = 419$ km$^2/T$, speed range 1.05-1.38 km/y.

Population E (Issongos) in Ref. (10): $D_N = 89$ km$^2/T$, speed range 0.48-0.64 km/y.

We may note that, for population E, almost all (81%) of individuals move less than 15 km (see its kernel above, section *Speed ranges predicted by the models*) and this leads to a low value of the demic diffusivity D_N and slow speeds. The error of ordinary diffusion is not negligible (e.g., for population E the fastest ordinary-diffusion speed implies an error of 30% relative to the more precise model above). However, the overall speed range (populations A-E) is $0.48 \leq s_{diff} \leq 1.58$ km/y under the approximation of ordinary diffusion. This is not very different from the range predicted by the more precise model above ($0.68 < s_D < 1.48$ km/y). More important errors due to ordinary diffusion arise when cultural transmission is included, as we now show.

Purely cultural model with ordinary diffusion. This corresponds to $D_N = 0$ in Eq. **S19**, i.e.

$$2\sqrt{\left(a_N + \frac{C}{T}\right) C\,D'}\ . \qquad \textbf{[S20]}$$

For each population, again the slowest speed is obtained for $a_N = 0.023\ y^{-1}$, $T = 35\ y$ and $C = 1$, and the fastest one for $a_N = 0.033\ y^{-1}$, $T = 29\ y$ and $C = 10.9$.

Population 1 (Mbuti, band I in Ref. (19)): $D' = 7.69$ km$^2/T$, speed range 0.21-2.17 km/y.

Population 2 (Mbuti, band II in Ref. (19)): $D' = 32.7$ km$^2/T$, speed range 0.44-4.48 km/y.

Population 3 (Mbuti, band III in Ref. (19)): $D' = 28.7$ km$^2/T$, speed range 0.41-4.20 km/y.

Population 4 (Aka (31)): $D' = 1.74$ km$^2/T$, speed range 0.10-1.04 km/y.

Population 5 (Baka (32)): $D' = 0.29\ \text{km}^2/T$, speed range 0.04-0.42 km/y.

We note that the under this approximation, the speeds of different populations differ by an order of magnitude (compare populations 2 and 5). This is much more than for the more precise model above. The overall range under the approximation of ordinary diffusion is 0.04-4.48 km/y, also much wider than for the more precise model above (0.03-0.66 km/y). Therefore, ordinary diffusion leads to very large errors in the purely cultural case. Thus in the main paper we have not applied ordinary diffusion (i.e., Fisher-type Eqs.) but the more precise, cohabitation-kernel model (i.e., Eq. **S11** above and Eq. **5** in the main paper).

For all populations 1-5, the ordinary-diffusion error is small for low values of C (slowest speed) but becomes very large when considering high values of C (e.g., for population 1 the ordinary-diffusion fastest speed is six times faster than for the more precise model above). This is due to the fact that cultural transmission has a non-local contribution (last term in Eqs. **S18**) and a local one (last-but-one term in Eqs. **S18**). The former is analogous to demic diffusion (first term on the right in Eqs. **S18**) but the latter has no demic analogue. This difference between demic and cultural diffusion comes from the computation of the change in the number density of farmers (Eq. **S17**). For demic diffusion, some of farmers arrive to the area considered (first term in the right of Eq. **S17**) and some leave it (second term in the right of Eq. **S17**). In contrast, for cultural diffusion convert farmers appear from hunter-gatherers (last term in Eqs. **S17**, which leads to the last two terms in Eqs. **S18**) but there is not a negative term in Eqs. **S17** because converted hunter-gatherers leaving the area considered do not imply any decrease in the *initial* number density of farmers. In fact, the local term (last-but-one term in Eqs. **S18**) has the usual form in reaction-diffusion equations, so it is not a property of cultural transmission only but of several-component reactive systems in general.

Demic-cultural model with ordinary diffusion. As explained above, the relevant result is its fastest speed. This is found by using Eq. **S19** with the value of D_N from the fastest demic case (population C), the value of D' from the fastest cultural case (population 2), the maximum intensity of cultural transmission ($C = 10.9$), the maximum initial growth rate (a_N =0.033 y^{-1}) and the lowest generation time (T=29 y). In this way, the maximum demic-cultural speed turns out to be 7.15 km/y for ordinary diffusion (Eq. **S7**). As expected from the previous paragraphs, this is much faster than the result from the more precise model above (3.04 km/y). Again this shows that ordinary diffusion (i.e., Fisher-type equations) cannot be trusted. Therefore, in the main paper we have applied the more precise model above.

S6. Effect of a short-range dispersal kernel. In the main paper, for simplicity we have applied a purely cultural model (Eqs. **S11** and **5**) in regions where agriculture spread more slowly than predicted by models with demic diffusion (i.e., the purely demic and the demic-cultural models in the main paper). The purely cultural model in the main paper neglects population dispersal, i.e., in that model individuals do not move at all. It may be argued that perhaps this is not a valid approximation, because many humans do not spend all of their lives at their birthplaces. The simplicity of this purely cultural model is somehow analogous to that of purely demic models, which disregard cultural diffusion but have been very useful during decades. The purely cultural model is similarly useful. Here we generalize it (at the expense of loosing simplicity) by taking

into account the dispersal of hunter-gatherers who have converted into farmers. In order to do so, consider a region where farming diffused culturally (i.e., without any incoming farmers). Imagine a boundary with farmers at one side and hunter-gatherers at the other side. Even if farmers do not cross the boundary to live and establish new farms at the other side, agriculture can still spread provided that some hunter-gatherers, living close enough to the boundary, learn agriculture and obtain domestic plants and/or animals from the farmers. These hunter-gatherers thus become (partially or totally) converted into farming. They can similarly teach agriculture to other hunter-gatherers, and this is sufficient to spread agriculture. This corresponds exactly to the model in the main paper (with final Eqs. **S11** and **5**). However, the converted hunter-gatherers and their children can also move to new locations and this will accelerate the spread of agriculture. It can be taken into account simply by replacing in Eqs. **S6, S8, S9** and **S10** the dispersal kernel of farmers $\phi_N\,(\Delta_x, \Delta_y)$ by the dispersal kernel of hunter-gatherers that have been converted into farmers, $\phi_N^{converts}(\Delta_x, \Delta_y)$. For example, Eq. **S6** is replaced by

$$N(x,y,t+T) = \int_{-\infty}^{\infty}\int_{-\infty}^{\infty} R_T\big[N\big(x+\Delta_x, y+\Delta_y, t\big)\big]\ \phi_N^{converts}\big(\Delta_x,\Delta_y\big)\, d\Delta_x\, d\Delta_y + \int_{-\infty}^{\infty}\int_{-\infty}^{\infty} c\big[N\big(x+\Delta_x, y+\Delta_y, t\big), P\big(x+\Delta_x, y+\Delta_y, t\big)\big]\ \phi_N^{converts}\big(\Delta_x,\Delta_y\big)\, d\Delta_x\, d\Delta_y. \qquad \textbf{[S21]}$$

Similarly, the final Eq. **S10** is replaced by

$$N(x,y,t+T) = \int_{-\infty}^{\infty}\int_{-\infty}^{\infty} R_T\big[N\big(x+\Delta_x, y+\Delta_y, t\big)\big]\ \phi_N^{converts}\big(\Delta_x,\Delta_y\big)\, d\Delta_x\, d\Delta_y + \int_{-\infty}^{\infty}\int_{-\infty}^{\infty} \phi_N^{converts}\big(\Delta_x,\Delta_y\big)\, d\Delta_x\, d\Delta_y\ \int_{-\infty}^{\infty}\int_{-\infty}^{\infty} \phi'_P\big(\Delta'_x,\Delta'_y\big)\, d\Delta'_x\, d\Delta'_y\ R_T\left[f\,\frac{N(x+\Delta_x+\Delta'_x, y+\Delta_y+\Delta'_y, t)\ P(x+\Delta_x, y+\Delta_y, t)}{N(x+\Delta_x+\Delta'_x, y+\Delta_y+\Delta'_y, t)+\gamma\ P(x+\Delta_x, y+\Delta_y, t)}\right]. \qquad \textbf{[S22]}$$

In order to compute the speed range predicted by this model, we need the dispersal kernel of hunter-gatherers that have been converted into farmers and their descendants, $\phi_N^{converts}(\Delta_x, \Delta_y)$. Both ethnographic (22,38-39) and genetic (40,41) observations indicate that partially converted HGs have substantially shorter dispersal distances than unconverted HGs or farmers. This implies that the kernel we need, $\phi_N^{converts}\big(\Delta_x, \Delta_y\big)$, has a shorter range than the dispersal kernels $\phi_N(\Delta_x, \Delta_y)$ above (populations A-E). Unfortunately, reliable estimates of the kernel $\phi_N^{converts}(\Delta_x, \Delta_y)$ are not available. There are genetic estimations (40) of its mean square distance (not of the whole kernel) but they disagree with ethnographic estimations (38). Verdu et al. (40) have proposed that this disagreement may be due to the fact that genetic estimates refer to distances between birthplaces of parents and children, whereas ethnographic estimates report distances between birthplaces and places of residence. In any case, the mean square distance cannot be applied because using a full kernel usually leads to a very different front speed than using only its mean square distance (10). Thus, in the absence of a reliable kernel $\phi_N^{converts}(\Delta_x, \Delta_y)$, we shall here approximate it to the short-range, cultural kernels $\phi'_P\big(\Delta_x, \Delta_y\big)$ used in the main paper. This seems reasonable as a first approximation, not only because both kernels have a short range but also because the cultural kernel gives the distances where hunter-gatherers learn farming, i.e. where they work in agricultural tasks. In fact, each hunter-gatherer usually performs agricultural work at several places, not at a single one (19), and he/she spends a substantial amount of time in such places. It can thus be expected that he/she may find his/her

mate at such a distance. For this reason, in this section we apply the cultural kernel $\phi'_P(\Delta_x, \Delta_y)$ as a first approximation to the dispersal kernel $\phi_N^{converts}(\Delta_x, \Delta_y)$. Of course, more precise results could be found if future work allows to measure both the cultural and the demic kernels for a given population of hunter-gatherers performing agricultural tasks. However, in the absence of both datasets for the same individuals, this seems a reasonable assumption in order to refine the purely-cultural model in the main paper. Then Eq. **S22** becomes

$$N(x,y,t+T) = \int_{-\infty}^{\infty}\int_{-\infty}^{\infty} R_T\left[N(x+\Delta_x, y+\Delta_y, t)\right] \phi'_P(\Delta_x,\Delta_y)\, d\Delta_x\, d\Delta_y + \int_{-\infty}^{\infty}\int_{-\infty}^{\infty} \phi'_P(\Delta_x,\Delta_y)\, d\Delta_x\, d\Delta_y \int_{-\infty}^{\infty}\int_{-\infty}^{\infty} \phi'_P(\Delta'_x,\Delta'_y)\, d\Delta'_x\, d\Delta'_y\, R_T\left[f \frac{N(x+\Delta_x+\Delta'_x, y+\Delta_y+\Delta'_y, t)\, P(x+\Delta_x, y+\Delta_y, t)}{N(x+\Delta_x+\Delta'_x, y+\Delta_y+\Delta'_y, t) + \gamma\, P(x+\Delta_x, y+\Delta_y, t)}\right], \quad \textbf{[S23]}$$

and Eq. **4** in the main paper is replaced by

$$s_{DC} = \min_{\lambda>0} \frac{a_N\, T + \ln\left[\left(\sum_{k=1}^{Q} P_k\, I_0(\lambda R_k)\right)\left(1 + C\left[\sum_{k=1}^{Q} P_k\, I_0(\lambda R_k)\right]\right)\right]}{T\lambda}. \quad \textbf{[S24]}$$

We use this equation to compute the speed ranges according to this model. For each population, again the slowest speed is obtained for $a_N = 0.023\ y^{-1}$, $T = 35\ y$ and $C = 1$, and the fastest one for $a_N = 0.033\ y^{-1}$, $T = 29\ y$ and $C = 10.9$.

Population 1 (Mbuti, band I in Ref. (19)): speed range 0.25-0.51 km/y.

Population 2 (Mbuti, band II in Ref. (19)): speed range 0.48-0.91 km/y.

Population 3 (Mbuti, band III in Ref. (19)): speed range 0.48-0.96 km/y.

Population 4 (Aka (31)): speed range 0.13-0.28 km/y.

Population 5 (Baka (32)): speed range 0.05-0.10 km/y.

Interestingly, these ranges are all similar to the corresponding ones for the purely cultural model. For example, for population 5 we have 0.05-0.10 km/y here versus 0.03-0.07 km/y (see the section above *Speed range from the purely cultural model*).

The overall range with the additional, short-range demic kernel is obtained by combining the 5 ranges above. This yields 0.05-0.96 km/y, similar to the range 0.03-0.66 km/y obtained for the purely cultural cohabitation-kernel model (see the section above *Speed range from the purely cultural model*).

We now need to address a subtle point. At first sight, we might be tempted to consider the ranges above as demic-cultural. However, this is not strictly correct because in this section population dispersal is due to the motion of hunter-gatherers who have converted into farming. This is not demic diffusion in the usual sense, which refers to populations of farmers that reproduce and disperse into new regions. Mathematically, the difference is that in proper demic diffusion the dispersal of individuals is computed by using a dispersal kernel of farmers, $\phi_N(\Delta_x, \Delta_y)$. In contrast, in the model in this section population dispersal is computed by using a dispersal kernel of converted hunter-gatherers $\phi_N^{converts}(\Delta_x, \Delta_y)$ (here approximated by their cultural kernel $\phi'_P(\Delta_x, \Delta_y)$). Therefore, we think that the model in this section should be more properly called a cultural model, rather than a demic-cultural model. In some sense there is

demic diffusion, but it is due to the motion of hunter-gatherers who have converted into farming (and their children, grandchildren, etc.). The genes of converted hunter-gatherers will be those of hunter-gatherers, not those of farmers (who have not reached this region). Similarly, the language (and other traits) of the population that is responsible for the spread of farming may also be that of their hunter-gatherer parents, grandparents, etc.

In the main paper, the red areas in Fig. 3 correspond to the purely cultural model (observed speeds 0.03-0.66 km/y). According to the more detailed model in this section, it could seem that those red areas should be replaced by regions with observed speeds 0.05-0.96 km/y. Note, however, that speeds within 0.68-0.96 km/y are also consistent with the demic-cultural model with mainly demic diffusion (0.68-1.36 km/y, see the main paper). Therefore, speeds within 0.68-0.96 km/y are consistent both with a cultural diffusion model (this section) and with a mainly demic model (main paper). They are thus shown as blue areas in Fig. S5. As expected, the mainly demic areas (yellow) are smaller in Fig. S5 than in Fig. 3 in the main paper, partially becoming additional areas where the dominant mechanism could have been either demic or cultural diffusion (blue). The main point is that the areas where we can safely conclude that cultural diffusion dominated are the same (red) in Fig. S5 as in Fig. 3. Similarly, the areas with too fast speeds to agree with our models (green) are also the same in Fig. S5 as in Fig. 3. As mentioned in our main paper, we think that the sea surrounding the British Islands may make the interpolation less reliable there, so our continental-scale interpolation approach should probably be modified for the British Islands (this local case could be analyzed in future work).

We have included Fig. S5 in this *SI Appendix* (rather than in the main paper) because Fig. S5 relies on the assumption that the dispersal kernel of converted hunter-gatherers is similar to their cultural kernel, but observations will be necessary to see whether this is a reasonable approximation or not (in the latter case, they will also provide precise dispersal kernels of converted hunter-gatherers, and it will be possible to use them into the model in this section).

If the purely cultural model in the main paper is replaced by the model in this section, Fig. 3 in the main paper is replaced by Fig. S5 in this *SI Appendix*. However, both figures lead to the following main conclusions. The Neolithic spread was cultural in Northern Europe, the Alps and West of the Black Sea (red, speeds below 0.66 km/y). On the other hand, it was mainly demic in other regions (yellow), except in regions where it was possibly either mainly demic or mainly cultural (blue, due to parameter uncertainty). The demic-cultural process (yellow/blue) was fast (speeds above 0.68 km/y) and includes a substantial part of the Linearbandkermic (LBK) culture in Central Europe (i.e., between the red region containing the Alps and the red region in Northern Europe in Fig. S5). Therefore, extending the purely cultural model in the main paper into the model in this section (i.e., by allowing for a short-range dispersal kernel of converted hunter-gatherers) does not change the main conclusions in the main paper.

Finally, we stress that some specific areas are uncertain because of they contain none sites of only a single one, e.g. the blue region in Croatia, some red regions in Spain, the non-red region in Belorussia, and the green areas in the continent (Fig. S5). These uncertain regions will likely disappear in the future, when additional sites are dated and are taken into account in the

interpolation maps. However, we think that additional sites are unlikely to change our main conclusions, because the density of sites is substantially higher in most regions of Europe.

S7. Justification of the definition of the cultural effect *E*. In the main paper we have defined the cultural effect by means of Eq. **6**, i.e. $E = \frac{s_{obs} - s_D}{s_{obs}} 100$, where s_{obs} is the observed speed at a given location in Europe (i.e., as estimated from the archaeological data) and s_D is the speed according to the purely demic model. Alternatively, we might be tempted to define the cultural effect as $\tilde{E} = \frac{s_C}{s_{obs}} 100$, where s_C is the speed according to the purely cultural model. Note that $\tilde{E} \neq E$ because $s_{DC} \neq s_D + s_C$ (see Eqs. **3**-**5** in the main paper). However $\tilde{E}$ does not provide a consistent estimation of the maximum cultural effect, for the following reason. Since the maximum value of s_C is 0.66 km/y (*Methods*), clearly $\tilde{E}_{max} = \frac{0.66}{s_{obs}} 100$. Thus in a region with, e.g., $s_{obs} = 0.80$ km/y, this would imply that cultural diffusion can be responsible for 0.66 km/y and demic diffusion for the difference, i.e. 0.14 km/y. But demic diffusion cannot cause an effect of only 0.14 km/y, since both s_D and s_{DC} are always ≥ 0.68 km/y (*Methods*). The intuitive reason is that the observed dispersal and reproductive behavior of preindustrial farmers implies a minimum Neolithic front speed (0.68 km/y) if there is demic diffusion of farmers. In contrast, the definition $E = \frac{s_{obs} - s_D}{s_{obs}} 100$, which has been previously applied (11), does not have this problem. The reason is that s_C can be as low as zero (in the purely demic model). Thus we can safely use the definition $E = \frac{s_{obs} - s_D}{s_{obs}} 100$, and the fact that $s_D \geq 0.68$ km/y, to estimate $E_{max} = \left(1 - \frac{0.68}{s_{obs}}\right) 100$ (as done in *Methods*). Thus in a region with, e.g., $s_{obs} = 0.80$ km/y, this implies that demic diffusion can be responsible for 0.68 km/yr and cultural diffusion for the difference, 0.12 km/yr. We conclude that using $\tilde{E}$ would be inconsistent with our results, whereas using E is not. In regions with $s_{obs} > 0.68$ km/yr, we have thus used E in the main paper (see Eq. **7** and the text below it), following previous work (11).

S8. Effect of dating uncertainties. As mentioned at the beginning of this *SI Appendix,* we used a dataset of 918 sites from Refs. (1,2). In that dataset, each archaeological site has a calibrated date and an error bar (namely, its reported date minus/plus its standard deviation). In order to see how this dating uncertainty affects the results, we sampled at random a value from each of the 918 error bars. Interpolating the thus-generated, new dataset, we obtained the isochrones in Fig. S6a. Comparing to the isochrones from the original dataset (Fig. S1a or Fig. 1), we see that they are very similar but not exactly the same (compare, e.g., the 7,500-yr isochrone in central Italy). We then applied the same smoothing technique as in the main paper (see the second section in this *SI Appendix*) to the new dataset. After smoothing 40 times, this yielded the isochrone map in Fig. S6b, which is again very similar to Fig. S4a (but not exactly the same). Applying the same procedure as in the main paper, we then obtained the speed map in Fig. S6c (the corresponding vector map is not shown because it is indistinguishable from Fig. S4c or Fig. 2). The speed map

from the new dataset (Fig. S6c) is similar to that from the original dataset (Fig. S4b or Fig. 3), but there are some differences. For the new dataset, the speed is somewhat slower in Western France (because the red region in Fig. S6c does not appear in Fig. S4b) and the British Islands. The same happens in the Iberian Peninsula and Croatia, but both areas contain very few sites and such regions cannot be trusted (as mentioned in the main paper and above). The main conclusions in the main paper are that the speed was (i) slower in Northern Europe, the Alpine area and West of the Black Sea (red color, cultural diffusion) and (ii) faster in the Balkans, Italy and a substantial part of the LBK culture in Central Europe (see the LBK map in Ref. (42), Fig. 12.7). Both conclusions hold also for the new dataset (Fig. S6c). We generated additional datasets, by sampling again a random value from each of the 918 error bars, and we always observed similar effects. Therefore, although dating uncertainties have noticeable effects, we have checked that they do not affect the main conclusions of our work. Let us finally stress that the latter also agree with the findings by some archaeologists and geneticists (see the main paper, *Discussion*).

1. Isern, N., Fort, J. & vander Linden, M. Space competition and time delays in human range expansions. Application to the Neolithic transition. *PLoS One* **7**, e51106 (2012).
2. Fort, J., Pujol, T. & vander Linden, M. Modelling the Neolithic transition in the Near East and Europe. *Amer. Antiq.* **77**, 203-220 (2012).
3. Burrough, P. A. & McDonnell, R. A. *Principles of Geographical Information Systems* (Oxford University Press, Oxford, 1998).
4. Conolly, J. & Lake, M. *Geographical Information Systems in Archaeology* (Cambridge University Press, Cambridge, 2006).
5. Oliver, M. A. & Webster, R. Kriging: a method of interpolation for geographical information systems. *Int. J. Geogr. Inf. Syst.* **4**, 313-332 (1990).
6. Piskunov, N. *Differential and integral calculus* (Mir, Moscow, 1966), Sec. 8.15.
7. ESRI, *ArcGIS 9. Using ArcGIS Spatial Analyst* (ESRI Press, Redlands, 2001). Available at http://downloads.esri.com/support/documentation/ao_/776Using_Spatial_Analyst.pdf (accessed 05/22/2013).
8. Ammerman, A. J. & Cavalli-Sforza, L. L. Measuring the rate of spread of early farming in Europe. *Man* **6**, 674-688 (1971).
9. Fort, J., Pérez-Losada, J. & Isern, N. Fronts from integrodifference equations and persistence effects on the Neolithic transition. *Phys. Rev. E* **76**, 031913 (2007).
10. Isern, N., Fort, J. & Pérez-Losada, J. Realistic dispersion kernels applied to cohabitation reaction-dispersion equations. *J. Stat. Mech.* **2008**, P10012 (2008).
11. Fort, J. Synthesis between demic and cultural diffusion in the Neolithic transition in Europe. *Proc. Natl. Acad. Sci. U.S.A.* **109**,18669-18673 (2012).
12. Stauder, J. *The Majangir: ecology and society of a southwest Ethiopian* people (Cambridge University Press, Cambridge, 1971).
13. Fort, J, & Méndez, V. Time-delayed theory of the Neolithic transition in Europe. *Phys. Rev. Lett.* **82**, 867-870 (1999).

14. Fort J, Pujol T Progress in front propagation research. *Rep. Prog. Phys.* **71**, 086001 (2008).

15. Fort J, Pérez-Losada J, Suñol JJ, Massaneda JM, Escoda L Integro-difference equations for interacting species and the Neolithic transition, *New J. Phys.* **10**, 043045 (2008).

16. Early, J. D. & Headland, T. N. *Population dynamics of a Philippine rain forest people. The San Ildefonso Agta* (University Press of Florida, Gainesville, 1998).

17. Fort, J., Jana, D. & Humet, J. M. Multidelayed random walks: Theory and application to the Neolithic transition in Europe. *Phys. Rev. E* **70**, 031913 (2006).

18. Wijsman, E. M. & Cavalli-Sfroza, L. L. Migration and genetic population structure with special reference to humans. *Ann. Rev. Ecol. and Systematics* **15**, 279-301 (1984).

19. Boyce, A. J., Küchemann, C. F., & Harrison, G. A. Population structure and movement patterns. In *Biological aspects of demography*, edited by W. Bras, pp. 1-9 (Barnes & Noble, New York, 1971).

20. MacDonald, D. H., & Hewlett, B. S. Reproductive interests and forager mobility. *Curr. Anthropol*. **40**, 501-523 (1999).

21. Fix, A. G., *Migration and colonization in human microevolution* (Cambridge Univ. Press, Cambridge, 1999), pp. 18 and 48.

22. Hewlett, B., van de Koppel, J. M. H., & Cavalli-Sforza, L. L. Exploration ranges of Aka pygmies of the Central African Republic. *Man* **17**, 418-430 (1982).

23. Cavalli-Sforza, Comments on 'Reproductive interests and forager mobility', Curr. Anthropol. **40**, 514-515 (1999).

24. Ammerman, A. J. & Cavalli-Sforza, L. L. *The Neolithic transition and the genetics of populations in Europe* (Princeton Univ. Press, Princeton, 1984).

25. Chagnon, N. A. *Yanomamö: the fierce people* (Rinehart and Winston, New York, 1968).

26. Bahuchet, S. Agricultural strategies as complementary activities to hunting and fishing. In *Food and nutrition in the African rain forest*, edited by C. M. Hladik, S. Bahuchet and I. de Garine (Unesco, Paris, 1989), pp. 31-35.

27. Cavalli-Sforza, L. L. & Bodmer, W. F. *Genetics of human populations* (Freeman, San Francisco, 1971).

28. Hewlett, B. & Cavalli-Sforza, L. L. Cultural transmission among Aka pygmies. *Amer. Anthropol.* **88**, 922-934 (1986).

29. Hart, J. A. *Nomadic hunters and village cultivators: a study of subsistence interdependence in the Ituri forest of Zaire.* Master of Arts Thesis (Michigan State University, East Lansing, 1979). Available at http://www.bonoboincongo.com/wp-content/uploads/2009/10/JAHartMastersThesis_1979.pdf (accessed 04/23/2014).

30. Hart, J. A. From subsistence to market: a case study of the Mbuti net hunters. *Human Ecology* **6**, 325-353 (1978).

31. Cavalli-Sforza, L. L. Demographic data. In *African Pygmies*, edited by L.L. Cavalli-Sforza, pp. 23-44 (Academic Press, New York, 1986).

32. Sato, H. Notes on the distribution and settlement patters of hunter-gatherers in Northwestern Congo. *African Study Monographs* **13**, 203-216 (1992).

33. Mulvaney, D. J. 'The chain of connection': the material evidence. In *Tribes and boundaries in Australia*, edited by Nicolas Peterson (Australian Institute of Aboriginal Studies, Camberra, 1976), pp. 72-94.

34. Boyd, R. & Richerson, P. J. *Culture and the evolutionary process* (University of Chicago Press, Chicago, 1985), p. 208.

35. Henrich, J. Cultural transmission and the diffusion of innovations: adoption dynamics indicate that biased cultural transmission is the predominant force in behavioral change, *Amer. Anthropol.* **103**, 992-1013 (2001).

36. Kandler, A. & Steele, J. Innovation diffusion in time and space: effects of social information and income inequality. *Diff. Fund.* **11**, 1-17 (2009).

37. Fisher, R. A. The wave of advance of advantageous genes. *Ann. Eugenics* **7**, 355-369 (1937).

38. Cavalli-Sforza, L. L. & Hewlett, B. Exploration and mating range in African pygmies. *Ann. Hum. Genet.* **46**, 257-270 (1982).

39. Cashdan, E. G//ana territorial organization. *Human Ecol.* **12**, 443-463 (1984).

40. Verdu, P., Leblois, R., Froment, A., Théry, S., Bahuchet, S., Rousset, F., Heyer, E. & Vitalis, R. Limited dispersal in mobile hunter-gatherer Baka pygmies. *Biol. Lett.* **6**, 858-861 (2010).

41. Henn, B. M., Cavalli-Sforza, L. L. & Feldman, M. W. The great human expansion. *Proc. Natl. Acad. Sci. U.S.A.* **109**, 17758-17764 (2012).

42. Kaczanowska, M. & Kozlowski, J. K. Origins of the Linear Pottery Complex and the Neolithic transition in Central Europe. In *The Widening Harvest. The Neolithic Transition in Europe: Looking Back. Looking Forward*, edited by A. J. Ammerman and P. Biagi, pp. 227-248 (Archaeological Institute of America, Boston, 2003).

Figure legends

Figure S1 │ Results of interpolating the dates of archaeological sites. (a) Isochrones with dates measured in calibrated years Before Present (cal y BP). (b) The corresponding local speeds, measured in km/y. (c) The corresponding orientations of the local speed vector (front propagation directions). In (a) and (b), the archaeological sites are shown as black dots. Compare to Fig. S4, where the same colors and speed ranges are used. Maps created with ArcGIS 10 and the Spatial Analyst extension.

Figure S2 │ (a) An enlarged portion of Fig. S1a shows the existence of small areas that are older than their surroundings (areas inside ellipses). They cause unrealistically small speed magnitudes and unrealistically abrupt changes in the speed orientation. This can be understood from an hypothetical example (b) if an older site (black dot in (c)) is added, causing an older region to appear in the isochrone map (8,000-y isochrone in (c)). The speed is slower and its direction changes more abruptly in (c) as compared to (b). In (a), the archaeological sites are shown as triangles.

Figure S3 │ Isochrones after smoothing a single time (a), 10 times (b) and 20 times (c). The archaeological sites are shown as black dots. Maps created with ArcGIS 10.

Figure S4 │ Isochrones after smoothing 40 times (a), the corresponding local speeds in km/y (b) and front propagation directions (c). In (a) and (b), the archaeological sites are shown as black dots. Compare to Fig. S1, where the same colors and speed ranges are used. Maps created with ArcGIS 10 and the Spatial Analyst extension.

Figure S5 │ Effect of a short-range dispersal kernel of converted hunter-gatherers in areas without incoming farmers. This figure is the same as Fig. 3 in the main paper, with the only difference that part of the yellow areas in Fig. 3 are blue here (indicating that either cultural or demic diffusion could have dominated). Map created with ArcGIS 10.

Figure S6 │ Effect of dating errors. Results from a dataset obtained by random sampling the date of each site from its error bar. Interpolation isochrones before (a) and after (b) smoothing. The local speeds (c) are given in km/y and have been obtained from the local slopes of the interpolated surface of Neolithic arrival dates after smoothing 60 times. The archaeological sites are shown as black dots. Maps created with ArcGIS 10 and the Spatial Analyst extension.
