# Supplementary figures and images for: Demic and cultural diffusion propagated the Neolithic transition across different regions of Europe
Source: J R Soc Interface. 2015 May 6;12(106):20150166. doi: 10.1098/rsif.2015.0166 (PMC4424695; doi:10.1098/rsif.2015.0166)

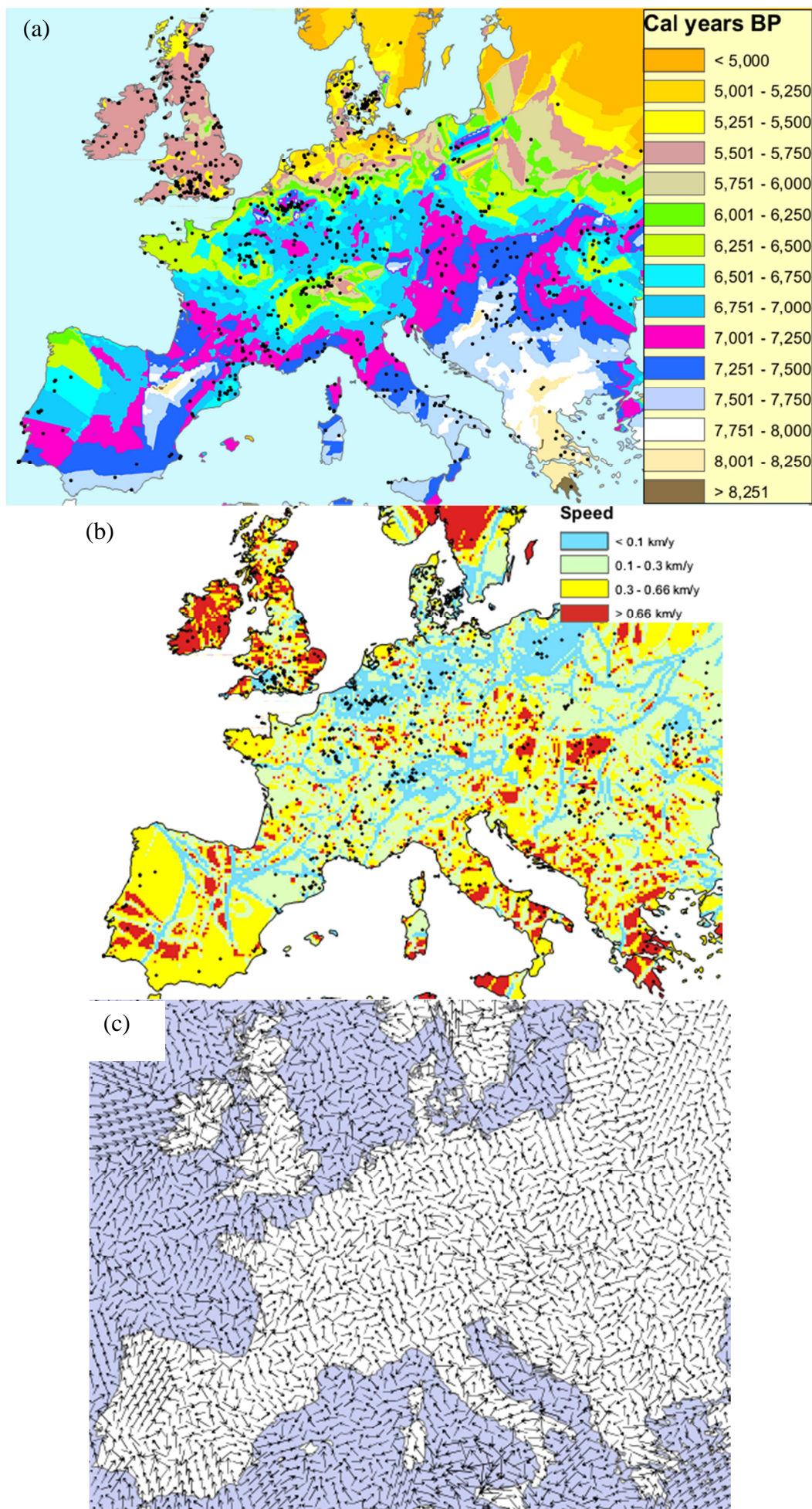

Fig. S1

Supplement: Fig. S1 [file rsif20150166supp2.pdf]

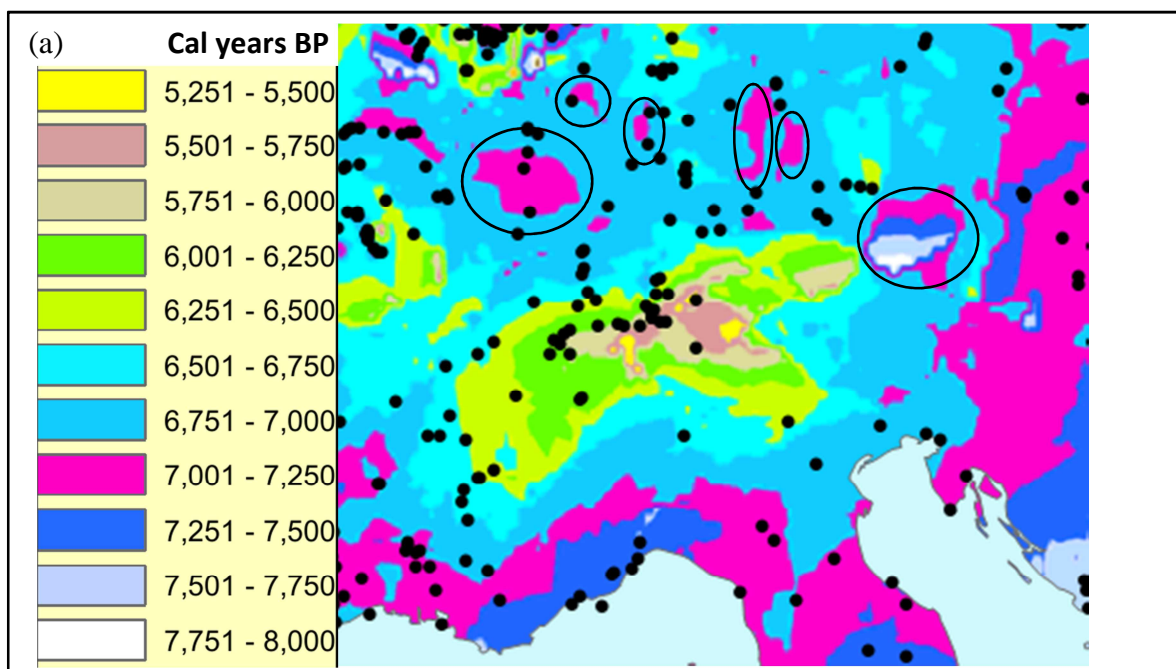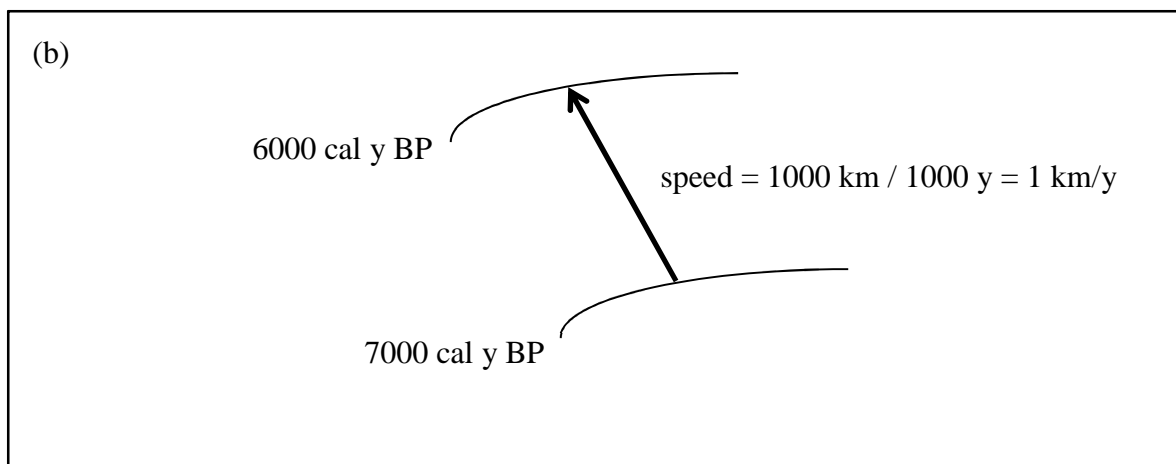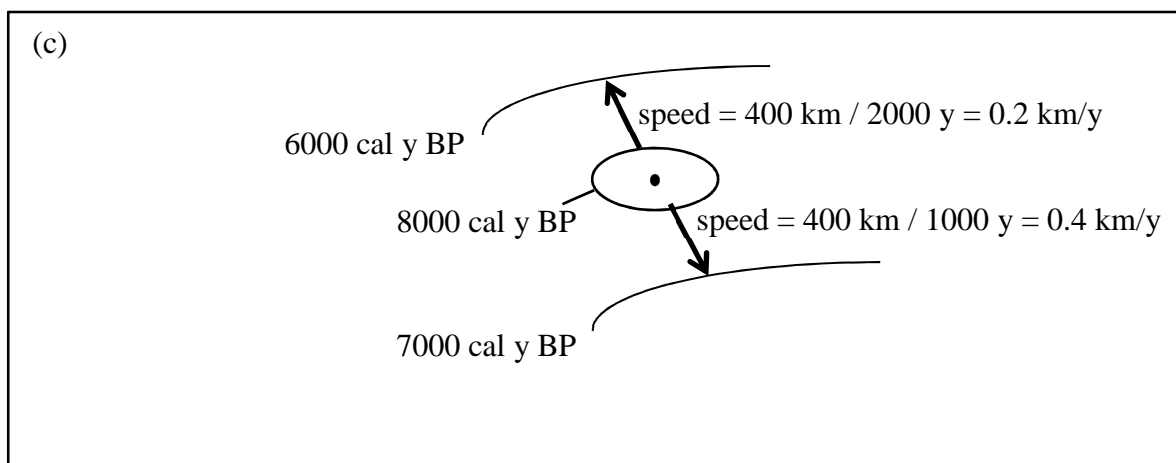

Fig. S2

Supplement: Fig. S2 [file rsif20150166supp3.pdf]

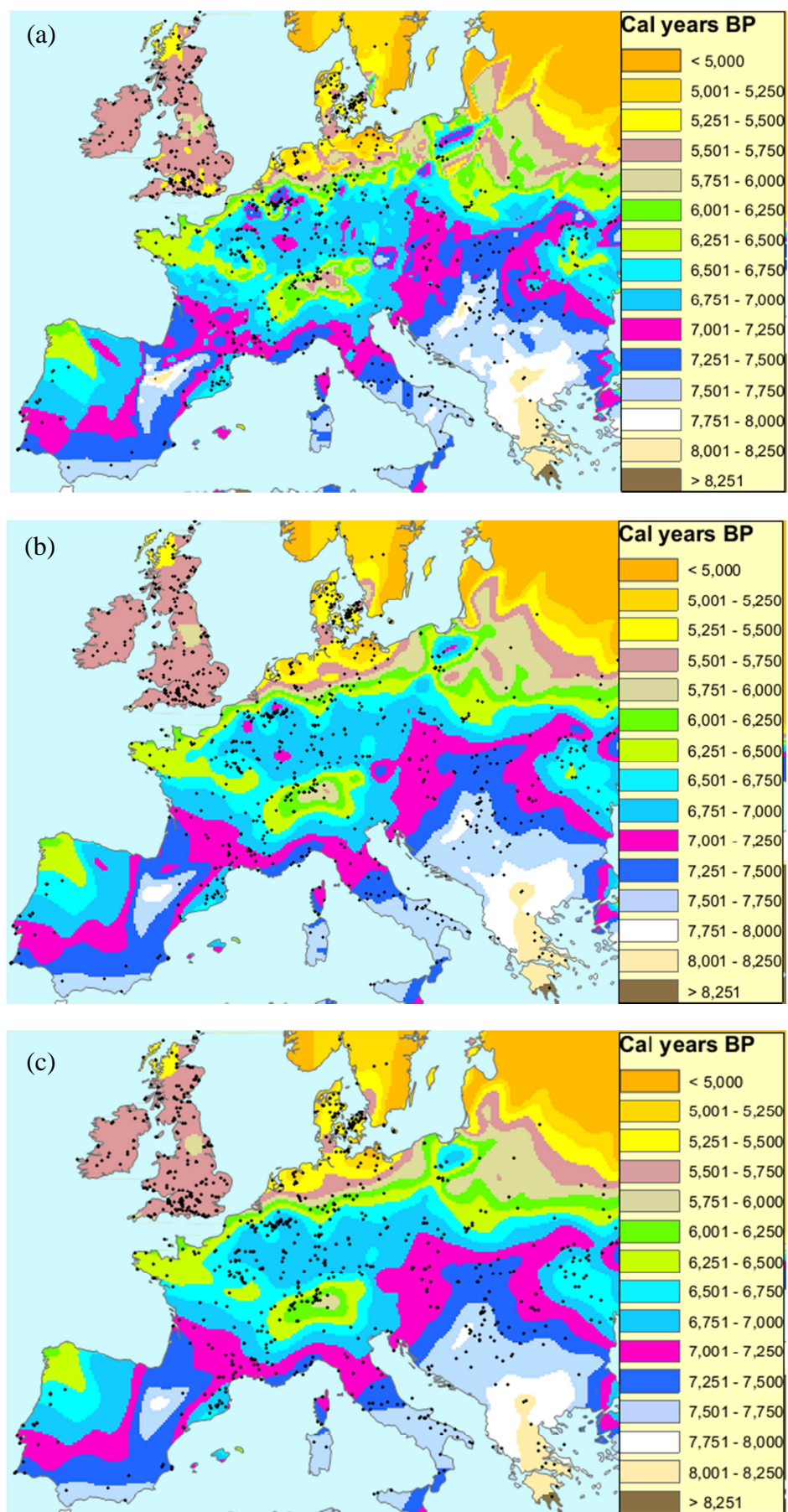

Fig. S3

Supplement: Fig. S3 [file rsif20150166supp4.pdf]

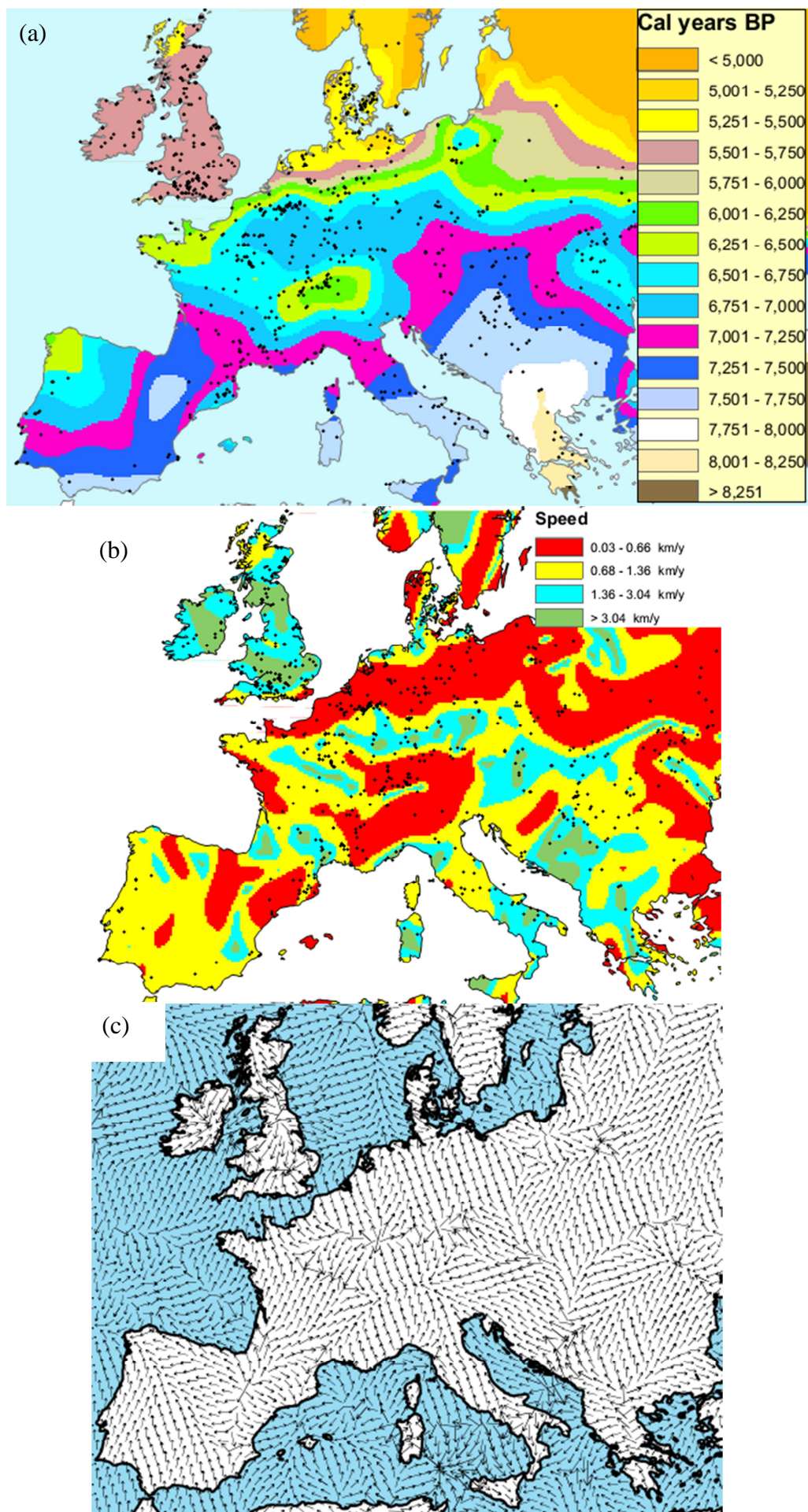

Fig. S4

Supplement: Fig. S4 [file rsif20150166supp5.pdf]

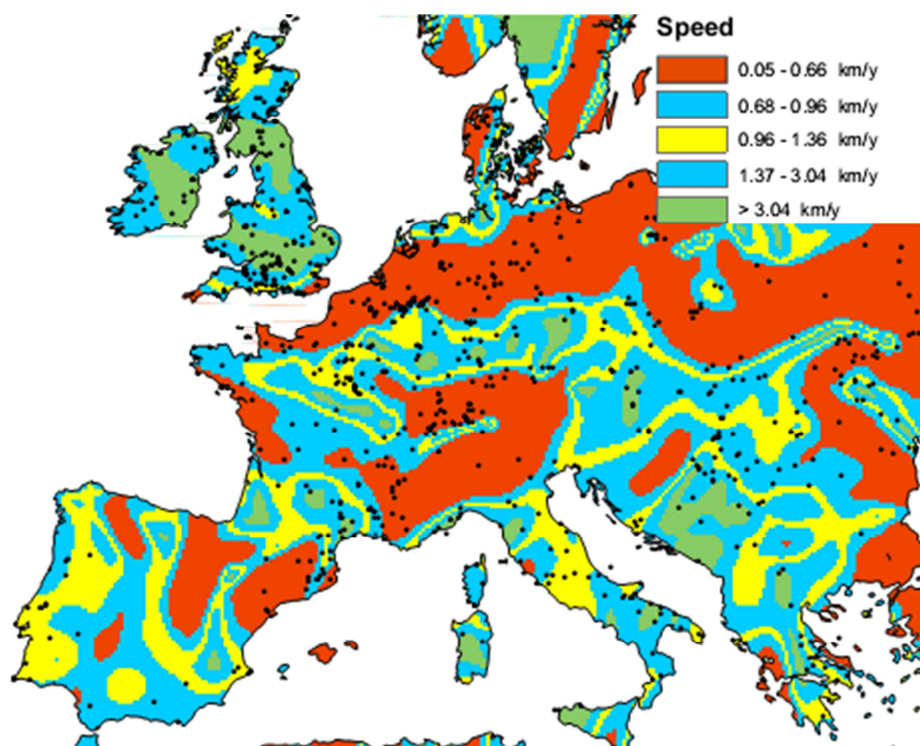

Fig. S5

Supplement: Fig. S5 [file rsif20150166supp6.pdf]

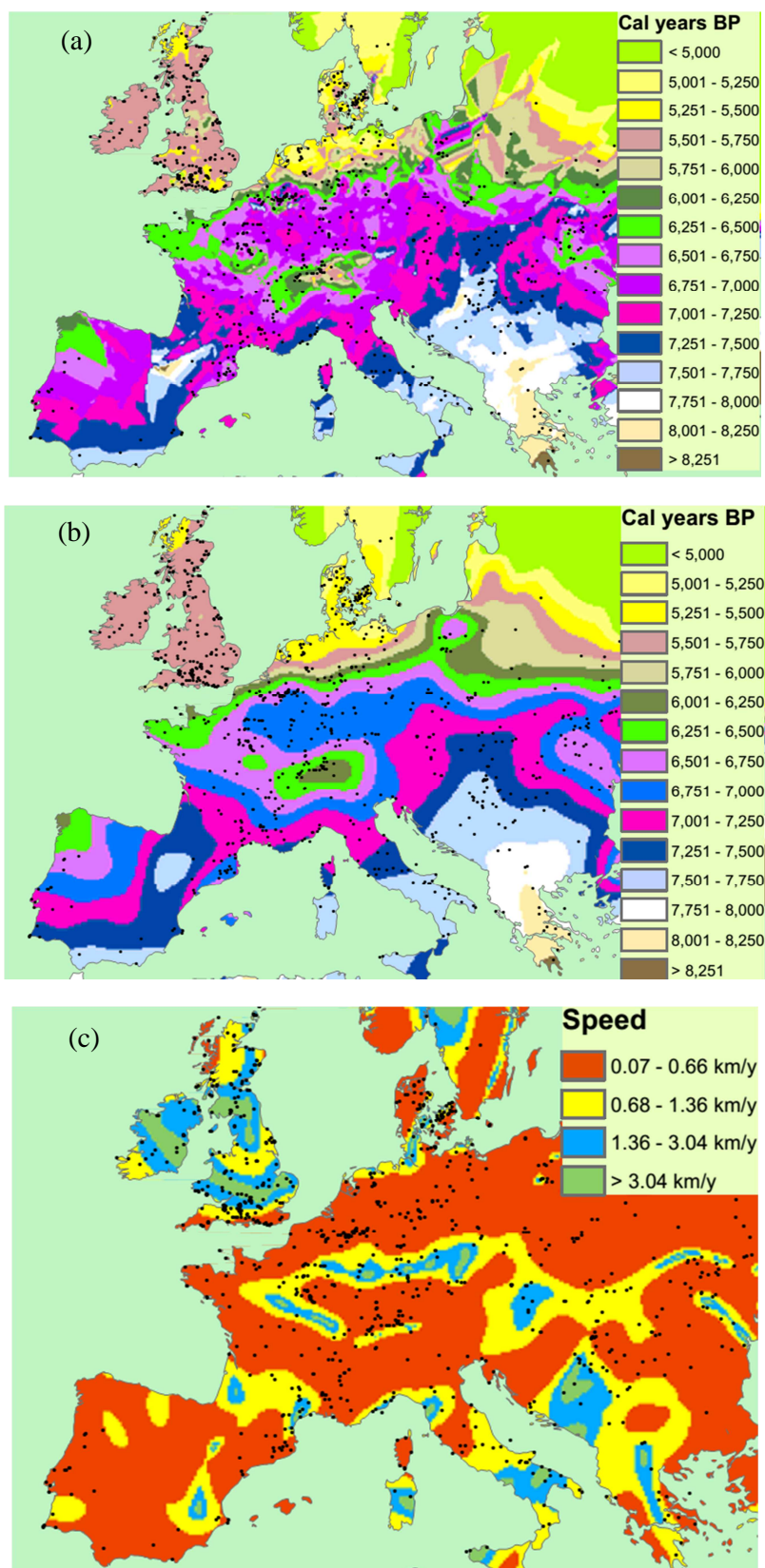

Fig. S6

Supplement: Fig. S6 [file rsif20150166supp7.pdf]
